# Systems Analysis of Drug-Induced Receptor Tyrosine Kinase Reprogramming Following Targeted Mono- and Combination Anti-Cancer Therapy

**DOI:** 10.3390/cells3020563

**Published:** 2014-06-10

**Authors:** Alexey Goltsov, Yusuf Deeni, Hilal S. Khalil, Tero Soininen, Stylianos Kyriakidis, Huizhong Hu, Simon P. Langdon, David J. Harrison, James Bown

**Affiliations:** 1 Scottish Informatics, Mathematics, Biology and Statistics Centre (SIMBIOS), Abertay University, Dundee, DD1 1HG, United Kingdom; E-Mails: y.deeni@abertay.ac.uk (Y.D.); H.Khalil@abertay.ac.uk (H.S.K.); 1305710@live.abertay.ac.uk (T.S.); J.Bown@abertay.ac.uk (J.B.); 2 The James Hutton Institute, Invergowrie, Dundee, DD2 5DA, United Kingdom; E-Mail: skyriakidis@live.com; 3 Lester and Sue Smith Breast Center, Baylor College of Medicine, One Baylor Plaza, Houston, TX 77030, USA; E-Mail: huhz03@gmail.com; 4 Division of Pathology, Institute of Genetics and Molecular Medicine, University of Edinburgh, Western General Hospital, Edinburgh, EH4 2XU, United Kingdom; E-Mail: Simon.Langdon@ed.ac.uk; 5 School of Medicine, University of St Andrews, St Andrews, KY16 9TF, United Kingdom; E-Mail: david.harrison@st-andrews.ac.uk

**Keywords:** HER2, HER3, trastuzumab, pertuzumab, combination cancer therapy, signalling reprogramming

## Abstract

The receptor tyrosine kinases (RTKs) are key drivers of cancer progression and targets for drug therapy. A major challenge in anti-RTK treatment is the dependence of drug effectiveness on co-expression of multiple RTKs which defines resistance to single drug therapy. Reprogramming of the RTK network leading to alteration in RTK co-expression in response to drug intervention is a dynamic mechanism of acquired resistance to single drug therapy in many cancers. One route to overcome this resistance is combination therapy. We describe the results of a joint *in silico*, *in vitro*, and *in vivo* investigations on the efficacy of trastuzumab, pertuzumab and their combination to target the HER2 receptors. Computational modelling revealed that these two drugs alone and in combination differentially suppressed RTK network activation depending on RTK co-expression. Analyses of mRNA expression in SKOV3 ovarian tumour xenograft showed up-regulation of HER3 following treatment. Considering this in a computational model revealed that HER3 up-regulation reprograms RTK kinetics from HER2 homodimerisation to HER3/HER2 heterodimerisation. The results showed synergy of the trastuzumab and pertuzumab combination treatment of the HER2 overexpressing tumour can be due to an independence of the combination effect on HER3/HER2 composition when it changes due to drug-induced RTK reprogramming.

## 1. Introduction

The ErbB receptor network consists of four members (ErbB1-4 or HER1-4): epidermal growth factor receptor (EGFR or HER1) together with human epidermal growth factor receptors HER2, HER3 and HER4. Although the ErbB receptor family belongs to the type I receptor tyrosine kinase (RTK) superfamily, the receptors HER2 and HER3 are non-autonomous and possess key defining features. HER2 has no known ligands and pseudo-kinase HER3 lacks tyrosine kinase activity [1]. These features define the interactions between HER2 and HER3 receptors for forming active heterodimer and homodimer complexes. These activation kinetics depend significantly on the expression levels of ErbB1-4 receptors and these levels vary across different cells. This combination of expression level variability and receptor interaction results in complex behaviour within the ErbB1-4 family.

For example, at normal expression levels HER2 functions as the shared co-receptor for EGFR, HER3, and HER4 receptors and these heterodimeric complexes are activated by the partner ligands. In contrast, overexpression of HER2 receptors in cancer cells causes constitutive activation of proliferation pathways in the absence of ligand through homodimerisation and RTK-driven auto-phosphorylation of HER2 [2,3]. Co-expression of various ligands of ErbB receptor contributes to both combinatorial complexity and redundancy in the ErbB network [4]. In this respect the ErbB network plays a role of communicator for the signals from at least six known ligands of EGFR receptors and neuregulins, ligand of HER3 and HER4, which represent a family of EGF-like growth factors encoded by at least four different genes, *NRG1-4* [5,6]. A higher level of complexity in ErbB signalling activity emerges due to the co-activation of different signalling pathways following RTK phosphorylation. Dimerization-driven stimulation of the intrinsic tyrosine kinase leads to the phosphorylation of tyrosine residues in the intracellular domain of the receptors that serve as docking sites to recruit a number of signal adapter proteins that link RTKs to different cellular signalling pathways such as phospholipase Cγ , PI3K/AKT/mTOR, MAPK, and STAT pathways [7,8].

Further complexity in ErbB activation and its signalling network arises from the complex regulation of co-expression of ErbB receptors and their ligands in the development of normal and cancerous tissue and organs. Complex and independent programs regulate expression of the different ErbB members and their ligands during morphogenesis in many organs. For example, in epithelial development ErbB receptors control epithelial differentiation and cell migration [6,9,10,11]. Expression of HER2 plays a significant role in adult heart function, where its mutations and deletion cause dilated cardiomyopathy in mutant mice [9]. Thus ErbB receptors and their ligands can act as transforming agents and complex regulators to generate a fully transformed phenotype [10].

In cancer, ErbB receptors and their ligands contribute to the generation of transformed malignant phenotypes [10]. Different co-expression profiles of ErbB receptors and EGF-like growth factors define different cancer types and subtypes [3,11,12]. This suggests that the progression, growth, migration, and survival of carcinoma cells are sustained, at least in part, by a network of RTK of the ErbB family and their ligands. Different co-expression profiles of the ErbB receptors and their ligands in human carcinomas lead to different tumour responses to drugs targeting the ErbB receptor system: from drug sensitivity to resistance, and—according to correlations in clinical data on ErbB1-4 co-expression profiles—disease-free survival and anti-RTK treatment outcome [13]. Amongst the range of inhibitors of RTKs, monoclonal antibodies have been developed to suppress dimerization of ErbB receptors. For example trastuzumab (Herceptin), a HER2 inhibitor, is an effective drug for HER2 overexpressing breast cancer [2,3,14].

RTK-targeted cancer therapies have been reported to fail when tumour cells circumvent the action of a single agent, and this therapeutic resistance is due to co-expression of different ErbB receptors in various cancer types [15]. Importantly, proteomic and genomic profiling revealed that this compensatory response to a single drug intervention results from a reprogramming of the RTK signalling networks following inhibition of one of the members of RTK family [16,17]. For example, transcriptional and post-transcriptional up-regulation of HER3 were reported to compensate for RTK-targeted therapy [18,19]. More recently, the development of combination therapies targeting multiple RTKs has been shown to successfully suppress *de novo* and acquired resistance [15], and the co-expression profile of different ErbB receptors is used as a basis for informed rational drug co-development and drug-diagnostics combination strategy [20,21]. In line with this strategy, some anticancer drugs have been approved and recommended for use in combination therapy. For example, pertuzumab, a humanized anti-HER2 monoclonal antibody, has been approved only in combination with trastuzumab [22]. In contrast to trastuzumab, pertuzumab mainly blocks ligand-dependent receptor heterodimerisation of HER2 and HER3 [23], while trastuzumab is more effective at ligand-independent tumour growth [24]. In this way pertuzumab effectively acts against tumours co-expressing HER2 (at low levels) and its other partners.

**Figure 1:**
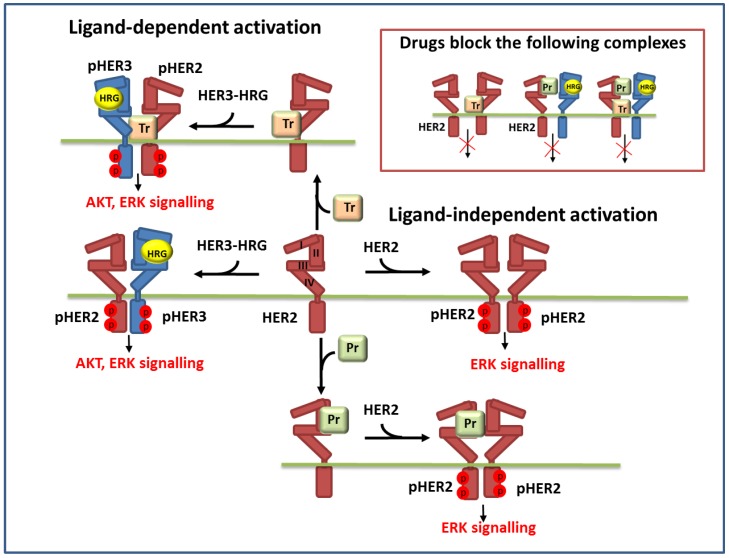
Schematic representation of the reactions of ligand-independent and ligand-dependent homo- and heterodimerisation of HER2 and HER3 receptors in the presence of HER3 ligand (HRG) and anti-HER2 drugs: trastuzumab (Tr) and pertuzumab (Pr). Inset in the upper right corner depicts the dimer complexes of HER2 and HER3 which are blocked by these drugs.

Trastuzumab and pertuzumab bind to different extracellular domains of HER2 and prevent both homo- and heterodimerisation of HER2 (Figure 1). This differential binding has been assumed to lead to a more effective inhibition when these drugs act in combination. Recent data show the synergistic antitumour activity of trastuzumab and pertuzumab combination therapy in HER2 overexpressing non-small cell lung, breast [25], gastric [26] and ovarian [27,28] cancers. The mechanisms of this observed synergistic combination effect are not yet clear. One proposed mechanism, based on the computational modelling of antibody interactions with HER2 receptors [29], is that the observed synergism might arise from cooperative binding of the two drugs with HER2 receptor and the formation of a ternary complex. Another mechanism of synergistic activity of trastuzumab and pertuzumab was reported in terms of the different actions of these two drugs: while trastuzumab inhibits the shedding of the extracellular domain (ECD) of HER2, which produces active truncated p95HER2 [28,30] and suppresses ligand-independent HER3/HER2 heterodimerisation [31], pertuzumab inhibits ligand-dependent HER2 heterodimerisation [14]. The combination of these actions leading to inhibitor synergism depends mainly on the co-expression of all the members of the ErbB family, their ligands, and receptor activating proteases. The benefit of this combination was suggested to correlate with up-regulation of HER2 heterodimerisation partners like HER3 and growth factors like heregulin during trastuzumab treatment [28].

To confirm this suggestion, and to design advanced combination therapeutic strategies generally, requires a deeper understanding of RTK co-expression regulation and its dynamical response to therapy. Computational systems biology offers a possible route forward to enhance our understanding of dynamical responses to therapy [32,33,34,35,36]. For example, recent systems biology research has proven useful in the study of the impact of complex RTK kinetics on cancer phenotypes and drug interaction with ErbB receptors of different expression levels [37]. The main attraction of computational modelling lies in the generation of kinetic models of different cancer phenotypes corresponding to a specific ErbB composition. The use of these models in describing drug action targeting RTKs facilitates the unravelling of the combinatorial contributions of different RTKs, co-expression levels of ErbBs and growth factors to drug effectiveness.

In a series of papers [38,39,40,41], computational models of the ErbB network were developed to establish the link between the complex kinetics of receptor dimerization and the integrated downstream signalling with consideration of ErbB and ligand co-expression in different cells. The following features of the ErbB receptor kinetics were established in joint *in vitro* and *in silico* experiments. First, HER2 has a diminished interaction with EGFR when HER3 was expressed in the cells [40]. Second, co-expression of EGFR and HER2-4 at low-to-moderate levels may enable a cell to match the phenotype of overexpression of HER2 due to the increasing dimerisation of HER3/HER2 [38]. Third, HER2 level is a strong determinant in phosphorylation of HER3 and attending HER3/PI3K/AKT signalling in the cells with different level of HER2 expression [39].

In this paper we describe a general approach to understand mechanisms of combination anti-HER2 drug effects in terms of reprogramming of the RTK signalling networks following mono- and combination therapies. To demonstrate this approach, we employ a computational systems biology approach to investigate the inhibition effects of pertuzumab and trastuzumab mono- and combination therapies in the context of changing levels of ErbB co-expression. We incorporate bioinformatic data on changes in gene expression profile during mono- and combination treatment [28] into our computational model which we then use to analyse the effectiveness of mono- and combination treatments. In particular, we investigate mechanisms of the trastuzumab and pertuzumab inhibition effects based on *in vitro* and *in vivo* experimental data on breast and ovarian cancer cell lines with a wide spectrum of ErbB co-expression.

## 2. Experimental

### 2.1. Computational Modelling

**Figure 2:**
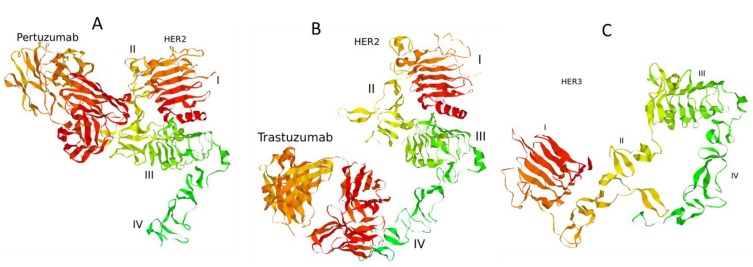
Complexes of the extracellular domain of HER2 receptor with pertuzumab (**A**) and trastuzumab (**B**). The structure of the extracellular domain of HER3 receptor (**C**). 3D structures of the proteins were retrieved from Protein Data Bank [43]: PDB codes 1S78 (**A**), 1N8Z (**B**), and 1M6B (**C**).

Model background: ErbB signalling network. In our model of ErbB signalling, we considered the kinetics of HER receptor activation and inhibition of HER2 by pertuzumab and trastuzumab alone and in combination (Figure 1). In the model we took into account heregulin (HRG)-induced HER3/HER2 heterodimerisation, which was reported to activate the most mitogenic signal and induce cellular growth in many cancers [12,24,42]. HRG binds with domains I and III of HER3 and changes the receptor conformations from “auto-inhibited” to “active” conformation resulting in a stretching of domain II, which is responsible for the formation of the receptor–dimer interface [3] (Figure 1 and Figure 2). This active conformation makes the receptor available for dimerization and stabilization of the receptor heterodimer with HER2 through an interaction between domain II in both receptors.

In contrast to HER3, the HER2 receptor has all four ECDs in a stretched conformation and this is assumed to result in uncontrolled dimerization and activation (Figure 2). Additionally, it has been assumed that the transmembrane region of HER2 contributes to self-association and activation control [3]. We considered ligand-independent homodimerisation of HER2 receptors at overexpression of HER2 (Figure 1).

The formation of HER2 homodimers and HER3/HER2 heterodimers stimulates RTK activity by tyrosine trans- and auto-phosphorylation. In the model we assumed that breast and ovarian cancer cells overexpressing HER2 can be activated in both a ligand-independent and dependent manners. When HER2 is overexpressed, RTK activation occurs ligand-independently because of constitutive HER2 homodimerisation; at normal expression levels, HER2 acts as the shared partner for other ErbB family members and these heterodimeric complexes are activated in response to the ligands.

**Figure 3:**
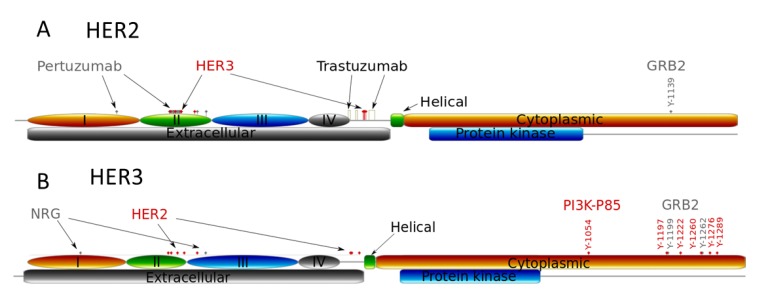
Schematic structures of the extra- and intracellular domains of receptors HER2 (**A**) and HER3 (**B**). Structures show the binding sites of pertuzumab, trastuzumab, HER2 and HER3, the receptor dimerization sites of the receptors, and the phospho-tyrosine binding sites with protein adapter Grb2 and PI3K (p85 subunit).

The tyrosine auto-phosphorylation sites in RTKs serve as docking sites for recruitment and activation of different adapter proteins that activate downstream responses induced by growth factor stimulation [6]. In Figure 3 we illustrate phospho-tyrosine docking sites to show the different interactions of HER2 and HER3 with both PI3K (p85 subunit) and Grb2, adaptor proteins of MAPK pathway. HER2 has only one interaction site with Grb2 and none with p85, the regulatory subunit of PI3K; HER3 has two binding sites for Grb2 and multiple sites for p85 binding [6]. This indicates that the homodimeric HER2 activates the MAPK pathway while the HER2/HER3 heterodimer activates both PI3K and MAPK pathways. Note that in the model we did not consider the indirect activation of PI3K/AKT signalling by HER2 through other cytosolic adapters [44]. For example, we neglected PI3K/AKT activation through binding GAB1 to Grb2 followed by PI3K (p85) binding and its activation induced by HER2 homodimerisation.

According to this difference in ECD structure of HER2 and HER3 receptors, changes in HER2 and HER3 homo- and heterodimerisation kinetics at various HER3/HER2 compositions mean that trastuzumab and pertuzumab can cause different inhibitory effects on PI3K and MAPK pathways despite targeting the same receptor, HER2. It follows that a change in the balance of homo- and heterodimerisation of HER2 and HER3 kinetics can impact the balance between PI3K/AKT and MAPK pathway activation. Further, pertuzumab binds primarily to the domain II of HER2, with a single binding to domain I [23], while trastuzumab binds in domain IV of the HER2 extracellular region [45]. The result of this differential binding is that these drugs block differently homo- and heterodimerisation of HER2.

We made use of the following experimental data to characterise how these two drugs change the activation kinetics of HER2/HER3 receptors. Pertuzumab efficiently inhibits ligand-induced HER3/HER2 heterodimerisation [31]. Trastuzumab does not block heterodimerisation of HER2 with ligand-activated EGFR or HER3 [2,46]. Pertuzumab blocks the association of HER2 with HER3 when cells are stimulated with HER3 ligand, HRG [14]. In the absence of HRG, the abilities of the antibodies to disrupt the ligand-independent HER3/HER2 complex were reversed: the amount of HER3 associating with HER2 was clearly reduced when cells were treated with trastuzumab.

We also took into account the cooperative interaction of trastuzumab and pertuzumab with HER2 receptors, which was investigated computationally in [29]. In Table 1 we summarize the action of trastuzumab and pertuzumab on homo- and heterodimerisation of HER2 discussed above.

In the model we neglected the formation of ligand-independent HER3/HER2 complexes that mainly occur when HER2 is overexpressed [31,47]. In this approximation we relied on the assumption that HRG/HER3/HER2 complexes could be more stable or abundant than the ligand-independent HER3/HER2 complexes [31]. Also, we did not consider the process by which trastuzumab prevents formation of p95HER2 (a truncated and constitutively active phosphorylated form of HER2 [28,30].

We applied this model to describe the effects of trastuzumab and pertuzumab on inhibition of ErbB signalling in SKOV3 ovarian cells with overexpressing HER2, a moderate level of EGFR, and low levels of HER3 and HER4 receptors [42]. We considered HER2 homodimerisation as a key promoter of RTK signalling and studied the impact of increasing HER3/HER2 heterodimerisation on RTK signalling when HER3 was up-regulated as a result of drug treatment. We neglected the impact of EGFR receptors in SKOV3 cells because experimental data suggest a dispensable role of EGFR in HER2 overexpressing cancers [24].

**Table 1:** Inhibition effects of trastuzumab and pertuzumab on homo- and heterodimerisation of HER3 and HER2 receptors in the presence/absence of HER3 ligands.

Drug	Receptor 1	Receptor 2	Ligand	Effect	Ref.
Trastuzumab	HER2	HER3	yes	no/minor	[46]
Trastuzumab	HER2	HER3	no	yes	[31]
Trastuzumab	p95HER2		no	yes	[30]
Trastuzumab	HER2	HER2	no	yes	[2]
Pertuzumab	HER2	HER3	yes	yes	[14]
Pertuzumab	HER2	HER3	no	no/minor	[31]
Pertuzumab	HER2	HER2	no	no	[2]

**Figure 4:**
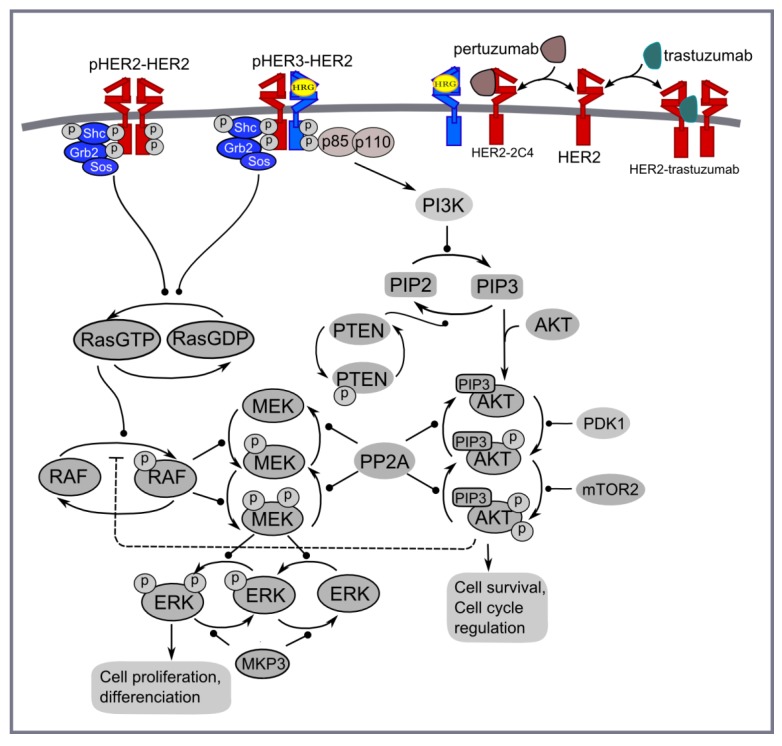
PI3K/AKT and ERK1/2 pathways activated by homo- and heterodimerisation of HER2 and HER3 receptors in the presence of HER3 ligand (HRG) and HER2 inhibitors: trastuzumab and pertuzumab.

*Computational model of pertuzumab and trastuzumab effect on MAPK and PI3K/AKT signalling*. The computational approach is based on the kinetic model of Ras/RAF/MEK/ERK and PI3K/PTEN/AKT signalling developed in [48]. This model describes the response kinetics of the signalling network to HRG-induced HER3/HER2 receptor heterodimerisation and the effect of HER2 inhibitor, pertuzumab (2C4 antibody), on ERK and AKT activation in the human ovarian carcinoma cell line PE04. The scheme of the signalling network is shown in Figure 4. The signalling network was parameterised by experimental data on the phosphorylation kinetics of HER2, ERK, AKT, and PTEN in the absence and presence of pertuzumab and was validated based on experimental data on the different combination effects of PTEN, PI3K, and HER2 inhibition [49,50,51,52].

We updated the model [48] to take into account homodimerisation of HER2 receptors that allows us to describe ligand-independent activation in HER2 overexpressing cancer cells and the inhibition effect of trastuzumab in HER2 overexpressing cancers (Figure 4). The parameters of the modules describing HER2 homodimerisation were adapted in such a way to represent the main impact of HER2 homodimerisation and HER3/HER2 heterodimerisation on signal activation at high and low HER2 concentrations, respectively (see discussion above). This modification of a previous version of the model significantly changes model behaviour at high concentrations of HER2 but does not affect model behaviour at HER3 and HER2 concentrations near their equimolar composition. The systems of ordinary differential equations corresponding to this updated model and the set of kinetic parameters are given in Supplementary Information 3.

### 2.2. Bioinformatics Method

We used microarray data on gene expression following trastuzumab (20 mg/kg), pertuzumab (20 mg/kg) and the combination treatment of SKOV3 tumour xenografts in mice [28]. In [28], gene expression data relating to proliferation, apoptosis, cell division and cell cycle were investigated and used to establish predictive biomarkers for trastuzumab and pertuzumab mono- and combination therapies. In this paper we focus on gene expression relating to RTK systems and downstream signalling pathways, namely PI3K/AKT and ERK1/2, which are modulated by the anti-HER2 therapies.

We made use of three different visualization methods to represent the results of statistical analysis of expression data. In addition to typical clustering of expression data in the form of heatmaps, we analysed both volcano plots and signalling network maps. Volcano plots represent fold change in gene expression level together with the statistical significance of this change [53]. Signalling network maps combine gene expression data with protein signalling network downloaded from the KEGG database [54]. We used the R programming language for statistical processing of the data (Student’s t-test) and Cytoscape [55] with CytoKEGG plugin for network illustration to integrate the gene expression data with the KEGG signalling networks.

### 2.3. Cell Lines and Treatment with Stimuli and/or Inhibitors

MCF-7 cells were obtained from ATCC while the HER2 over-expressing cell line, MCF-7/HER2–18 cell line, was a kind gift from the Osborne/Schiff laboratory, Baylor College of Medicine, USA. Both cell lines were routinely maintained in Dulbecco’s Modified Eagle Medium (DMEM) containing phenol red supplemented with 10% foetal calf serum (FCS, HarlanSera-Lab LTD) in a 37 °C incubator with 5% CO_2_. Cells were grown in DMEM without phenol red containing 5% double charcoal stripped serum (DCSS) for 48 h before antibody or HRG-β1 (1 nM) treatment. Cells were incubated with trastuzumab (100 nM) or pertuzumab (100 nM) for 20 min prior to HRG-β1 treatment. For the MCF7/HER2 cell line, the medium was supplemented with 500 μg/mL of Geneticin (Invitrogen). Cell lysates were prepared as previously described [48]. Phospho-Akt and phospho-ERK were measured by reverse phase protein array (RPPA). Treated samples were denatured and reduced protein lysates were spotted onto nitrocellulose-coated glass slides (Eurogentec, Hampshire, UK). Three replicates were spotted per sample in eight two-fold dilutions. Slides were hydrated in Whatman wash buffer for 5 min, Li-Cor Odyssey blocking buffer for 1 h (LI-COR Biosciences, Nebraska, USA), and then incubated with primary antibodies overnight at 4 °C in a sealed box containing a damp paper towel. The following day slides were washed in PBS/T at room temperature for 5 min (×3) before incubating with far-red fluorescently-labelled secondary antibodies diluted in Li-Cor Odyssey Blocking Buffer (1 μL/2 mL) at room temperature for 45 min with gentle shaking. Slides were then washed in excess PBS/T (×3)/PBS (×3) and allowed to air dry before reading on a Li-Cor Odyssey scanner at 680 nm and 780 nm and images exported as TIFF files for further analysis. Antibodies used were anti-pERK1/2 (T202/Y204) (Rabbit, Cell Signalling Technology), anti-pAKT (S473) (Rabbit, Cell Signalling Technology), anti-GAPDH (Rabbit, Cell Signalling Technology) and anti-GAPDH (mouse, Abcam). The secondary antibodies were IRDye 800CW goat anti-mouse or rabbit and IRDye 680 goat anti-mouse or rabbit. The signals were captured with an Odyssey scanner (Li-COR) for fluorescence measurement. RPPA analysis was performed using MicroVigene RPPA analysis module (VigeneTech, Carlisle, MA, USA). Spots were quantified by accurate single segmentation, with actual spot signal boundaries determined by the image analysis algorithm. Each spot intensity was quantified by measuring the total pixel intensity of the area of each spot (volume of spot signal pixels), with background subtraction of 2 pixels around each individual spot. The mean of the replicates was used for normalisation and curve fitting. Curve fitting was performed using five-parameter logistical non-linear regression using a joint estimation approach (“supercurve method”). The quantification y0 (intensity of curve) of sample dilution curves was normalised by corresponding total protein.

## 3. Results and Discussion

### 3.1. Computational Modelling of Mono- and Combination Anti-HER2 Therapies at Different Co-Expression Levels of HER3/HER2 Receptors

**Figure 5:**
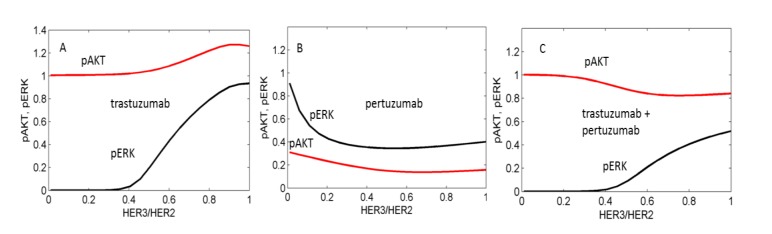
Computational results of the effects of trastuzumab (**A**), pertuzumab (**B**), and their combination (**C**) on pAKT (red lines) and pERK (black lines) signals depending on the ratio HER3/HER2. pAKT and pERK signals are normalized to their values in the absence of drugs. Minimum concentration of HER3 is 5 nM.

We used the computational model to calculate the inhibition of pAKT and pERK by pertuzumab and trastuzumab alone and by their combination in the presence of HRG and analysed the dependence of the inhibition level on the ratio of expression of HER3 to HER2 (Figure 5). Results revealed that trastuzumab alone inhibits pERK at a low ratio of HER3/HER2, which corresponds to HER2 overexpression (Figure 5A). In the model this effect is consistent with HER2 homodimerisation causing ERK activation but not AKT activation (see model description in Methods). Therefore trastuzumab blocking HER2 homodimerisation suppresses pERK signalling but does not affect pAKT signalling. With an increase in HER3 expression, inhibition of pERK by trastuzumab decreases because trastuzumab does not block an increasing heterodimerisation of HER2 with an active HER3 which signals to pERK. The additional activation of pAKT observed in Figure 5A at increased HER3 expression is also due to increasing HER3/HER2 heterodimerisation signalling to pAKT.

In *in silico* experiments, pertuzumab was observed to effectively inhibit both pERK and pAKT signalling when HER3/HER2 > 0.1 (Figure 5B). At HER2 overexpression (HER3/HER2 < 0.1) pertuzumab does not inhibit pERK because it does not block HER2 homodimerisation, and only inhibits pAKT signalling activated by HER2 heterodimerisation.

The drug combination effectively inhibits pERK at lower ratios of HER3/HER2 due to blocking of HER2 homodimerisation by trastuzumab and causes an inhibition effect of up to 60% at HER2/HER3 > 0.5 (Figure 5C). In our calculation the drug combination does not markedly inhibit pAKT in this region of high HER3/HER2 levels. We suggest that the presence of the two HER2 inhibitors leads to an increase of free HER3 receptors that stimulates HER3/HER2 heterodimerisation at a high concentration of HER2 and suppresses pAKT inhibition by the combination.

### 3.2. Genetic Reprogramming of HER2/HER3 Signalling Following Anti-HER2 Therapy

**Figure 6:**
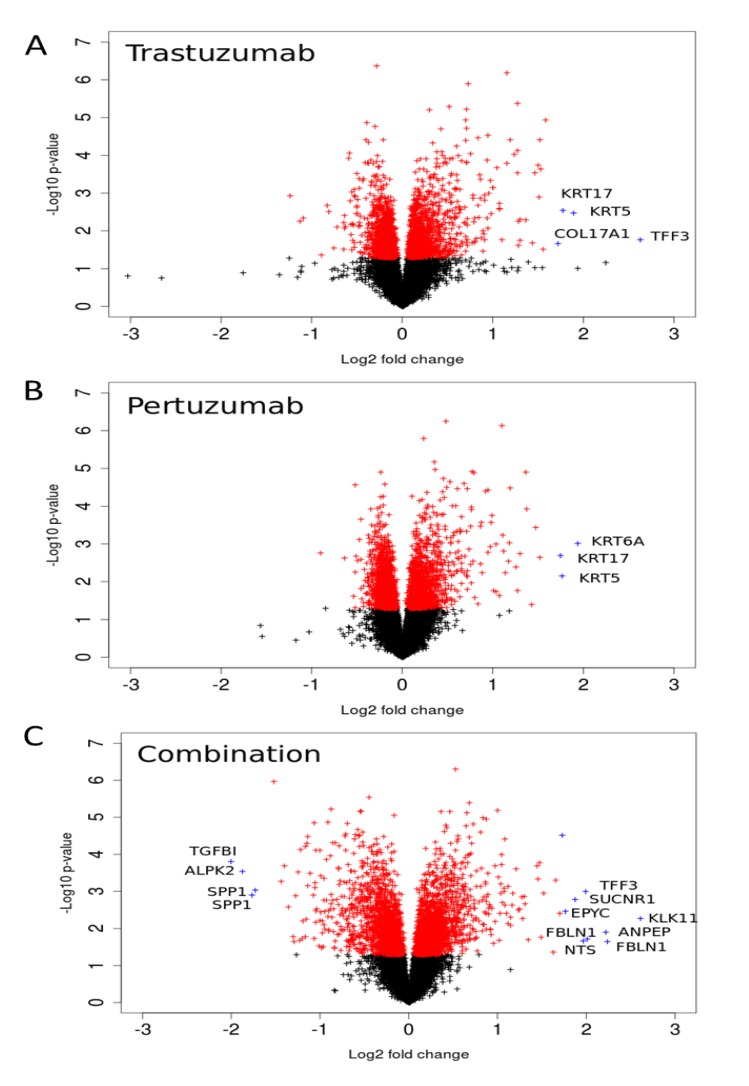
Volcano-plots of gene expression following trastuzumab (**A**), pertuzumab (**B**), and their combination (**C**) treatment of SKOV3 ovarian xenograft tumour [28]. The genes with statistically significant (*p*-value < 0.05) and non-significant fold changes are coloured by red and, black, respectively. Genes with a high fold change are marked by blue crosses.

We analysed gene expression data on the response of SKOV3 xenograft tumour to trastuzumab, pertuzumab, and their combination treatment [28]. The volcano plots illustrated in Figure 6 indicate genes that have high expression level changes and where that change is significant. Many of the genes that were differentially expressed in the combination group were already modulated by trastuzumab monotherapy with the combination treatment producing an even greater effect, especially in terms of down-regulation (Figure 6). However there were also gene expression changes that were modulated differentially between the combination and monotherapy treatments suggesting more complex behaviour. Genes that were more markedly expressed in the drug combination group included the secreted glycoprotein *FBLN1, EPYC* (which regulates fibrillogenesis), *ANPEP*, *SUCNR1*, *KLK1*, *TFF3*, and *NTS* (the full names of genes are given in Supplementary Information 1). Genes down-regulated by the combination include *TGFBI*, *ALPK2*, and *SPP1.* For both monotherapy treatments, keratin genes were more highly expressed after either pertuzumab (*KRT5*, *6A*, and *17*) or trastuzumab treatment (*KRT5*, *17*) than combination treatment. Similarly, *COL17A1*, a collagen gene, was also increased more after monotherapy than combination treatment (2.5-fold and 2.2-fold with trastuzumab or pertuzumab respectively *vs.* 1.2-fold for the combination treatment).

**Figure 7:**
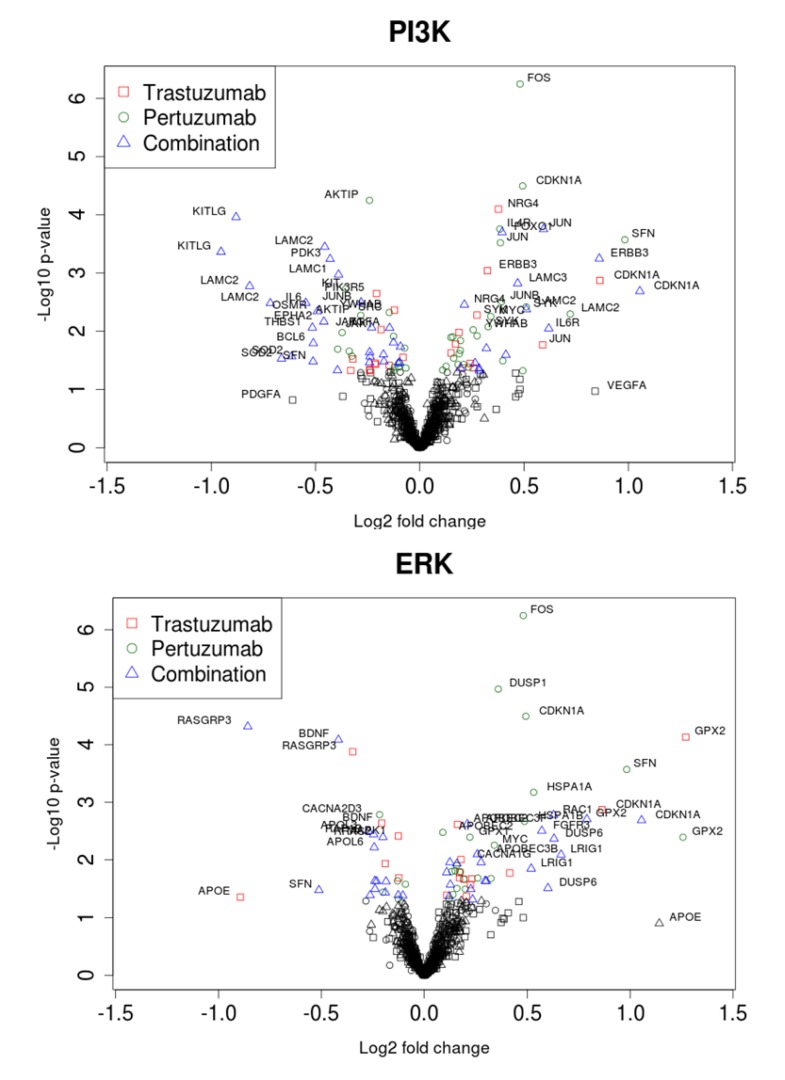
Volcano plots of gene expression related to PI3K/AKT (**A**) and ERK1/2 (**B**) pathways during trastuzumab, pertuzumab, and their combination treatment of SKOV3 ovarian xenograft tumour [28].

**Figure 8:**
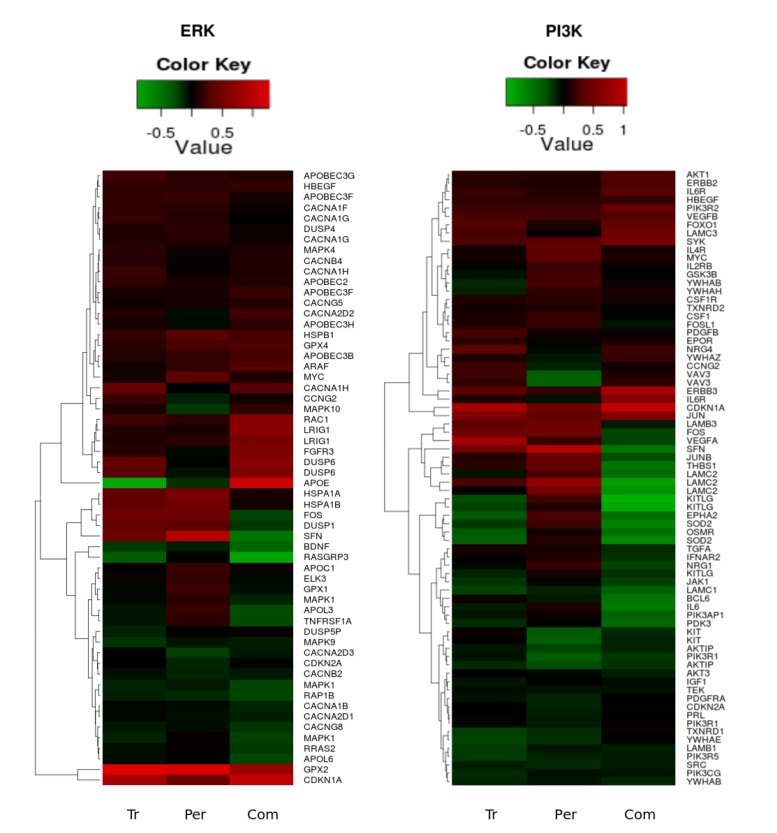
Heatmaps of the significant (*p*-value < 0.05) gene expression fold change in the PI3K/AKT (left heatmap) and ERK1/2 (right heatmap) pathways following trastuzumab (Tr), pertuzumab (Per), and their combination (Com) treatments of SKOV3 ovarian xenograft tumour [28].

We sought to follow the action of drugs on HER2 and HER3 receptors and the resultant outcomes (cellular growth inhibition) by narrowing our focus to the ErbB network and its signalling feeds into the ERK1/2 and PI3K/AKT pathways. The expression data on the genes encoding proteins of these pathways and the dependent genes under control of AKT and ERK signalling are represented in volcano plots and heatmaps in Figure 7 and Figure 8, respectively. Pronounced effects of the drugs were observed at the receptors levels: *HER3* expression was increased 1.8-fold in the combination group and by 1.3 and 1.1-fold with trastuzumab and pertuzumab, respectively. Expression of *HER2* receptor barely changed (1.1, 1.1, and 1.2-fold in trastuzumab, pertuzumab and combination groups, respectively). Among genes encoding HER3 ligands only *NRG4* was changed noticeably (1.3, 1.0, and 1.2) in this regard.

The genes encoding signalling proteins of PI3K/AKT and ERK1/2 pathways underwent weak perturbations under drug treatments: expression changes are noticeable only for *AKT1* (1.1, 1.1, and 1.2) and *PIK3R2* (1.2, 1.2, and 1.3).

Other receptors with expression level increases after combination treatment included *FGFR3* and *IL6R* and the GTP binding molecule *RAC1*. Downstream components in the PI3K/AKT pathway included *JUN* and *CDKN1A*. Among the genes encoding receptors and proteins connected to the PI3K/AKT network through different cross talk interactions, there was notable and significant up-regulation of expression of *FOXO1*, *HBEGF*, *FGFR*, *VEGFB*, *SYK*, *LAMC3*, *RAC1*, *JUN*, and *CDKN1A*, accompanied by significant down-regulation of expression of *TGFA*, *IGF1*, *EPHA2*, *PDGFRA*, *JAK1*, *SRC*, *TEK*, *LAMB1*, *LAMC1*, and *PDK3*, most especially following combination treatment (see Figure 8).

Within the set of the genes encoding proteins connected to the ERK1/2 network, there was significant up-regulation of *HSPB1*, *APOBEC2*, *APOBEC3B*, *APOBEC3F*, *APOBEC3H*, *CACNG5*, *CACNA1H*, *MAPK4*, *ARAF*, and *LRIG1*, and significant down-regulation of *RASGRP3*, *RAP1B*, *APOL6*, *CACNA1B*, *CACNG8*, and *YWHAB* following combination therapy. It is of interest to note that there were drug combination specific and significant differential down-regulation changes in the expression of *FOS*, *JUNB*, *THBS1*, *LAMC2*, *LAMB3*, *VEGFRA*, *IFNAR2*, *SFN*, *DUSP1*, *IL6*, *BCL6*, *IL1A*, *TGFA*, *APOL3*, *APOL6*, *RRAS2*, and up-regulation of *APOE* and *MAPK10* (see Figure 8).

Figure 8 reveals clusters in both of the pathways where all treatments cause a clear up-regulation in the genes *HER3*, *FOXO1*, *PIK3R2*, *GPX2* and *CDKN1A.* It was interesting to note that several genes, such as *CDKN2A*, *KITLG*, *KIT*, *OSMR*, *SOD2*, *GPX1, DUSP6*, *FOXO3*, *MAPK1*, *MAPK10*, *GSK3B*, *ELK3*, *FOSL1*, *LAMC3*, *DUSP4*, *MYC*, *APOL3*, *EPHA2*, *CACNA1H*, *CACNA2D2*, *CACNA2D3*, *VAV3*, *YWAHZ*, *YWHAB*, *NRG4*, *NRG1*, *TNFRSF1A*, and *IL6* were specifically and differentially expressed in similar fashion following trastuzumab or combination (Figure 8 and data in Supplementary Information 1). These are genes within the PI3K/AKT, ERK1/2 and FOXO networks, as well as genes associated with calcium homeostasis, cellular proliferation, growth arrest, differentiation, adhesion, motility and migration.

**Figure 9:**
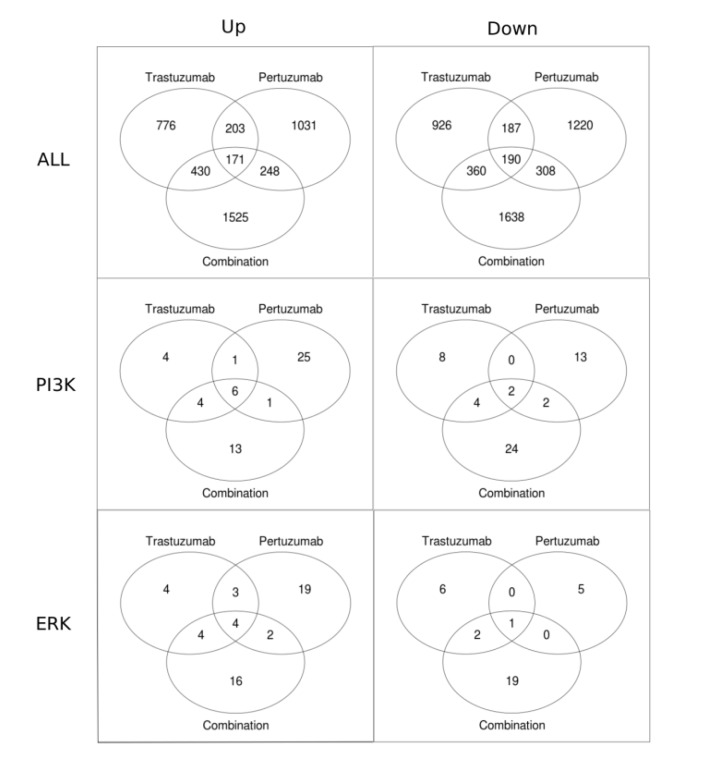
Venn diagrams showing the numbers of genes with statistically significant fold change in trastuzumab, pertuzumab, and their combination treatments of SKOV3 ovarian xenograft tumour [28]. The first row of the figures contains all the genes up-regulated (left column) and down-regulated (right column). The second and the third rows show Venn diagrams for the genes associated with the PI3K/AKT and ERK1/2 pathways, respectively.

Venn diagrams in Figure 9 present the numbers of genes that have a statistically significant fold change for different treatments. Figure 9 shows that the total number of genes with a statistically significant fold change is substantially higher (approximately 2-fold) in the combination treatment than in either the trastuzumab or pertuzumab group. Also shown is that for each of the treatments the total number of genes that are significantly down-regulated is greater than that significantly up-regulated. Focusing on the PI3K/AKT and ERK1/2 pathways revealed the opposite trend: more genes are up-regulated than down-regulated. This difference in reprogramming magnitude and direction between global (all data) and local (specific pathway data) may be attributable to cross-talk between the PI3K/AKT and ERK1/2 pathways together with the contribution of other compensatory mechanisms and pathways in attaining the overall globally more balanced reprogramming observed (the first row in Figure 9 and Figure 6), especially with the combination therapy.

To visualise reprogramming of ErbB signalling network as a result of a change in gene–gene interaction networks in response to drug treatments we integrated gene expression data into the signalling map of the ERK1/2 and PI3K/AKT pathways (Figure 2 in Supplementary Information 2). In this signalling diagram, the nodes (proteins) are coloured according to the log2 fold change in expression of corresponding genes at the different treatments.

### 3.3. Linking the Efficacy of Mono- and Combination Therapy to ErbB Co-Expression Level

**Figure 10:**
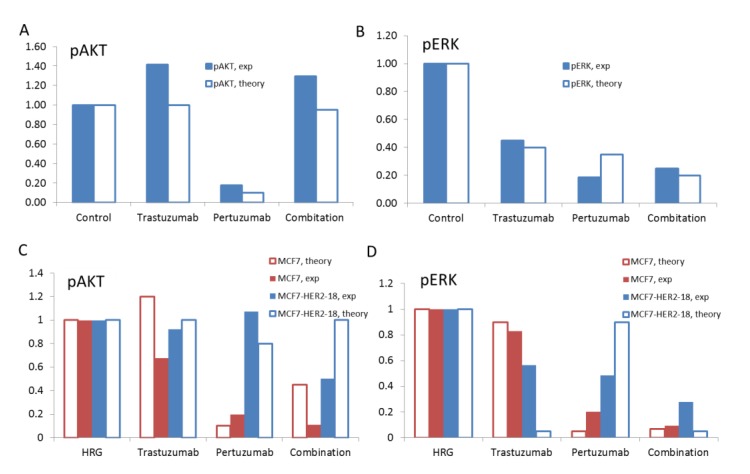
The theoretical results on the effects of trastuzumab, pertuzumab, and their combination on pAKT (A) and pERK (**B**) at the ratio HER3/HER2 = 0.5 and pAKT (**C**) and pERK (**D**) at the ratio HER3/HER2 = 0.1 (blue bars) and 1 (red bars). Comparison of the computational (open bars) and experimental (filled bars) data for pAKT (**A**) and pERK (**B**) in SKOV3 ovarian xenograft tumour after 14 days [28] and pAKT (**C**) and pERK (**D**) in MCF7 and MCF7-HER2-18 cells [57,58] treated by trastuzumab, pertuzumab, and their combination.

We compared model results of the inhibition effects of pertuzumab and trastuzumab with the measurements of pAKT and pERK signalling in SKOV3 xenograft tumours after 14 days of treatment with pertuzumab and trastuzumab individually and in combination [28] (Figure 10A and B). We considered the effect of drug-induced reprogramming of signalling pathways on the effectiveness of these drugs. As reported in [28] and discussed above, pertuzumab and trastuzumab and their combination induced different signalling regulations at the gene level. More pronounced changes were observed for genes encoding ErbB receptors and especially HER3. The expression of genes encoding signalling proteins of the downstream PI3K/AKT and ERK1/2 pathways changed less. In line with this experimental data, we considered only alteration of the expression level of receptors, HER2 and HER3, and neglected changes in protein levels within PI3K/AKT and ERK1/2 pathways in the model. This strategy also can be justified by the results of sensitivity analysis of the models of PI3K/AKT and ERK1/2 pathways which revealed ErbB receptors as the more sensitive module and signalling proteins were ranked the next highest sensitive subsystem in the signalling model [35,51,52,56].

In the model we suggested that the up-regulation of HER3 leads to a change in the HER3/HER2 composition in the SKOV3 xenograft tumour relative to its initial ratio in the SKOV3 cell line [42]. The increasing role of the HER3 receptor during tumour growth and treatment is also evident in the experimental data on phospho-HER3 measured in the SKOV3 tumour xenograft at day 14 [28]. In the *in vitro* experiments, for untreated SKOV3 cells pHER3/pHER2 ratio is less than 0.1 [42], whereas in the SKOV3 xenograft tumour at day 14 the ratio of pHER3/pHER2 is approximately 3 for untreated, 1 for trastuzumab, 0.3 for pertuzumab, and 1.4 for drug combination treatment [28]. In the calculation, we also considered the expression of the ligand of HER3 receptor, HRG4, which was observed to be expressed in the SKOV3 tumour xenograft (Figure 8).

To compare our computational results with the *in vivo* data we took the ratio of total proteins HER3/HER2 in the range of 0.5 assuming an increase of HER3/HER2 composition in HER2 overexpressing SKOV3 cells as a result of the up-regulation of HER3 during drug treatment. At this ratio the model satisfactory describes experimental data for 1) the effective inhibition of pERK and 2) the absence of inhibition of pAKT at trastuzumab treatment alone (Figure 10A and B). As discussed above, the absence of pAKT inhibition by trastuzumab is due to the fact that trastuzumab targets HER2 homodimerisation that mainly signals to the ERK1/2 pathway but not the PI3K/AKT pathway. This is consistent with reports supporting trastuzumab-mediated inhibition of both pERK and cell growth in HER2-overexpressing breast cancer cell lines [2,59].

Comparison of *in silico* and *in vivo* experimental results on the effect of pertuzumab showed that pertuzumab alone inhibits effectively both pAKT and pERK at a HER3/HER2 ratio in the range of 0.5. As HER3/HER2 dimers are a key target of pertuzumab and HER3 was reported to be biomarker of pertuzumab effectiveness [24,42], the inhibition of the HER3/HER2 activation signals by pertuzumab may confirm the observed increase of the HER3 expression in SKOV3 cells following the pertuzumab treatment.

Our computational results on the effect of the combination treatment agree with experimental data in respect of a strong inhibition of pERK and no effect on pAKT as measured in the SKOV3 tumour xenograft (Figure 10A and B). As discussed above, in the model, pAKT is not inhibited by the combination as a result of increasing AKT activation at increasing HER3 level in the presence of high concentration of HER2 in SKOV3 cells (see Section 3.1).

In this comparative analysis of *in silico* and *in vivo* experimental data, we suggested an increase of HER3/HER2 composition from low level (HER2 overexpression) up to 0.5 that occurs in SKOV3 cells as a result of the drug treatment. In this transient region between HER2 overexpressing and equimolar receptor composition, trastuzumab is still effective and the capability of pertuzumab to inhibit HER3/HER2 signalling becomes noticeable. To validate our model we applied it to describe drug effects at the boundary HER3/HER2 composition: low (HER2 overexpression) and equimolar levels, and then compared theoretical results with experimental data. To exclude the effect of alterations in receptor composition due to treatment we considered *in vitro* experimental data on MCF7 and HER2 overexpressing MCF7-HER2-18 cells [57,58].

To adapt our model to describe MCF7 cells we used the following experimental data on HER3/HER2 composition. A ratio of HER3 mRNA to HER2 mRNA was reported to be equal approximately to 0.3 [60] while the HER2 transfectants, MCF7/HER2-18 cells was reported to have 45-fold overexpressing HER2 receptors on the cell surface [61] and a ratio of mRNA HER3/mRNA HER2 = 0.03 [60]. Based on this data we assumed the equimolar composition of HER3/HER2 for MCF7 and 0.1 for MCF7-HER2-18 cells. We also assumed lower HER2 concentration in MCF7 cells than in HER2 overexpressing SKOV3 cells (see receptor concentrations in the model given in Table 3 in Supplementary Information 3). Note that some of the observed discrepancy between computational and experimental results is likely to be due to uncertainty in experimental data on surface receptor concentrations of different cells.

Computational results and corresponding experimental data on pAKT and pERK inhibition by trastuzumab, pertuzumab and their combination in HRG-activated MCF7 and MCF7-HER2-18 cells are shown in Figure 10C and D. The theoretical results agree with experimental data in respect of a weak ability of trastuzumab to inhibit both pAKT and pERK in MCF7 cells with an equimolar composition of HER3/HER2 (Figure 10C and D). We suggest that uninhibited pAKT and pERK signals correlate with the inability of trastuzumab to inhibit the growth rate of MCF7 cell under HRG stimulation [57]. At HER2 overexpression, trastuzumab does not inhibit pAKT according to both theoretical and experimental data in MCF7-HER2-18 cells. Trastuzumab inhibits pERK by almost 100% in the model but by only 40% in the experimental data. Experimental data on the growth of MCF-7/HER2-18 induced by 1 nM and 0.1 nM HRG showed that 100 nM trastuzumab slowed down the growth rate [57].

Pertuzumab effectively inhibits both pAKT and pERK signalling in MCF7 cells in the experiment and theory. In HER2 overexpressing MCF7/HER2-18 cells pertuzumab is unable to suppress pAKT according to both experimental and computational results and inhibits pERK by 50% (10% in the model). The drug combination inhibits effectively both pAKT and pERK in MCF7 cells. In the MCF7/HER2-18 cells the combination inhibits effectively pERK and pAKT by 50% (no inhibition in the model).

The comparison of *in vitro* and *in vivo* data along with our computational results allowed us to conclude that *in vivo* mono- and combination treatments show features common to both MCF7 and MCF7/HER2-18 cells which possess significantly different HER3/HER2 compositions. This might confirm our assumption on the transient status of HER3/HER2 composition in SKOV3 under anti-HER2 treatment. According to both experimental and computational results, the combination treatment shows a composition independent inhibition effect on pERK signalling: the drug combination effectively inhibits pERK at both high HER2 and HER3/HER2 equimolar compositions (see last bars in Figure 10D). In contrast, the effects of either monotherapy are composition dependent. This observation allows us to speculate on the mechanism of synergy of trastuzumab and pertuzumab combination inhibition of pERK signal in a wide range of HER3/HER2 composition. We suggest that this drug synergism maintains the high effectiveness of the combination in *in vivo* experiments on inhibition of SKOV3 xenograft tumour in spite of a dynamically changing HER3/HER2 composition during treatment [28].

### 3.4. Discussion

Genetic reprogramming of signalling networks is a general phenomenon following drug cancer therapy that leads to therapeutic resistance via activation of alternative, compensatory signalling pathways to cell proliferation and survival. Up-regulation of RTK and kinome networks following corresponding targeted therapy are convincing evidence for this drug-induced reprogramming phenomena [16,17,18]. Recent experimental and clinical data show that drug combinations with pronounced synergetic action effectively inhibit cancer cell proliferation and cause tumour regression in many cancers despite signalling reprogramming [16,62,63].

In RTK networks, genetic reprogramming leads to a change in relative expression levels of the ErbB receptors and their ligands. Here, we focused on the relationship between expression levels of HER2 and HER3 receptors which are observed to impact significantly on cancer cell phenotype, response to anti-HER2 therapy [24,42], and drug resistance [17,18,19]. The main mechanism underlying this phenomenon appears to be activation of compensatory circuits allowing cancer cells to by-pass the drug intervention. The same phenomenon could be one mechanism of resistance to trastuzumab therapy in HER2 overexpressing cancers [15]. An observed HER3 up-regulation suggests that HER2 activity alone is not sufficient to drive cancer cell growth [8,11], and sensitivity to HER2-targeted therapy correlates with HER3 expression in HER2 overexpressing breast cancer cell lines [2,24,42]. Also, the importance of HER2 heterodimers in tumour cells has been demonstrated in xenograft studies, where the combination treatment of trastuzumab and pertuzumab resulted in potent antitumour effects in HER2 overexpressing breast and ovarian cancers [8,28].

We analysed gene expression modulation following trastuzumab, pertuzumab and their combination in SKOV3 ovarian xenograft tumours overexpressing HER2 [28]. Each therapy caused significant reprogramming of many signalling pathways (see volcano plots in Figure 6). We observed differences in the sets of genes significantly up- and down-regulated in response to trastuzumab, pertuzumab and their combination, and these differences may be caused by the following mechanisms. First, trastuzumab and pertuzumab can have effects on targets other than HER2 that are specific to these drugs and this could result in different genetic responses (see introduction). Second, these differences could result from differential effects of the two drugs and their combination on activation of the downstream pathways of RTK signalling: PI3K/AKT and ERK1/2 [28]. As evident from *in vitro*, *in vivo*, and *in silico* experimental data discussed above, trastuzumab, pertuzumab, and their combination differently inhibit pAKT and pERK signals and thus differently modulate transcription of various genes being under their control. The specific genetic response of the cells to the treatments and the distinct inhibition of PI3K/AKT and ERK1/2 signalling pathways by the two drugs alone and in combination define different inhibition of tumour growth rate: pertuzumab slowed down tumour growth; trastuzumab resulted in strong growth inhibition; and the combination caused stronger growth inhibition of SKOV3 tumour xenograft [28].

We observed that the shape of volcano plot depends on drug treatment (Figure 6): the shape of volcano plot for the combination treatment is symmetric while mono-therapies give distinct asymmetries in the shape with respect to up- and down-regulated genes. Asymmetries could be due to either: 1) an imbalance in a number of up- and down-regulated genes; or 2) weaker down-regulation in comparison to up-regulation of genes in the case of the individual drugs. To distinguish between these possibilities we estimated the number of up- and down-regulated genes for all treatments. The numbers of down- and up-regulated genes are approximately the same, *i.e.* their imbalance is less than 6%, which is unlikely to impact the symmetry of the volcano plots. The main reason of the transformation from an asymmetric to a symmetric shape with the combination therapy is therefore due to a stronger down-regulation that causes the “eruption” on the left-hand side of the volcano plot (Figure 6C). We suggested that the reason for this pronounced down-regulation is likely to be due to stronger inhibition of pERK by the drug combination observed in the SKOV3 xenograft tumour [28].

To explore this possible reprogramming of the RTK system, we used computational modelling to investigate the response of PI3K/AKT and ERK1/2 pathways to pertuzumab and trastuzumab mono- and combination therapy depending on the dynamics of HER receptor co-expression levels. We incorporated bioinformatics data on gene expression following drug treatments in to the computational model. The main factor considered in the model was the ratio of HER3 to HER2. Based on analysis of gene expression data, we suggested that initially low HER3/HER2 composition in HER2 overexpressing SKOV3 cells increases at the later stage of the treatment as a result of HER3 up-regulation, and especially for the combination treatment. In the computational model we considered that up-regulation of HER3 receptors reprograms ErbB dimerization kinetics from ligand-independent HER2 homodimerisation to ligand-dependent HER3/HER2 heterodimerisation and this then becomes the predominant receptor signalling complex due to up-regulation of the HER3 ligand and autocrine shedding of HRG4 ligand (Figure 8).

Based on our modelling we suggested that trastuzumab alone strongly inhibits tumour growth at the initial stage of treatment at low HER3/HER2 ratio by suppressing pERK, and trastuzumab retains its ability to inhibit pERK at higher HER3/HER2 ratios in the range up to 0.5 at the later stage of treatment when the HER3/HER2 ratio increases (Figure 5 and Figure 10). Assuming that ERK1/2 activation is a growth driver of HER2 overexpressing SKOV3 tumour, this inhibition effect drives SKOV3 tumour growth inhibition [28]. In contrast, pertuzumab effectively inhibits both pAKT and pERK only at the late stage of the treatment when HER3 is up-regulated and fails to inhibit pERK signalling at low HER3/HER2 ratio at the beginning of the treatment. This may be one of the reasons that pertuzumab only slowed down tumour growth [28]. The combination of trastuzumab and pertuzumab effectively inhibit pERK proliferation signalling at the initial stage of SKOV3 xenograft tumour growth because trastuzumab in combination suppresses ligand-independent homodimerisation of overexpressing HER2 receptors and continues to cause inhibition of pERK at the later stage of the treatment when the HER3/HER2 composition increases as a result of HER3 up-regulation. At this later stage pertuzumab effectively inhibits ligand-dependent HER3/HER2 heterodimerisation which becomes the predominant receptor signalling complex activating both pERK and pAKT pathways. We thus suggested that the synergy in the combination treatment of HER2 overexpressing tumour is due to each drug acting effectively at different stages of tumour treatment. The effectiveness of this combination is assumed to link to RTK reprogramming in HER overexpressing cancer. HER3 up-regulation following RTK-targeted therapy was observed in many cancer cases and was shown to lead to resistance to single drug therapy [18,19,24,62,64]. Thus the investigation of the response of HER3 to different drug therapy and the mechanisms of its up-regulation can help to design more effective combination anti-RTK therapy.

HER3 up-regulation as a consequence of cell reprogramming following monotherapy has been observed in several studies. Increased HER3 cell surface expression leading to resistance to inhibition of RTK signalling by Gefitnib has been attributed to signalling network reprogramming [65]. In HER2 overexpressing breast cancer cell lines, inhibition of the PI3K pathway was observed to lead to up-regulation of HER3 gene expression that in turn transmitted downstream signals to pAKT, thus abrogating a sustained PI3K inhibition [66]. Further, PI3K inhibition led to up-regulation of transcripts of a number of other RTKs and their phosphorylated forms, e.g. HER3, IGF-1R, InsR, EphA1, and FGFR [64] demonstrated the tendency of HER2 overexpressing cancer cells to engage compensatory pathways for sustained growth and malignancy.

These observations are consistent with previous reports addressing similar pathways. Chandarlapaty *et al.* [67] used a wide panel of human cancer cell lines to show similar up-regulation of HER3 following AKT inhibition. They attributed this to a negative feedback loop between PI3K and RTK via Forkhead box transcription factor (FOXO): the PI3K/AKT pathway normally keeps transcriptional activity of the FOXO family of transcription factors in check by phosphorylation that leads to their sequestration in the cytoplasm [68]. It was reported that following AKT [67] or PI3K [64] inhibition FOXO re-localised to the nucleus where it functioned as a transcription factor for RTKs thereby up-regulating their transcription. In our results, the transcription of *FOXO1* was significantly up-regulated by all therapies (Figure 8). Additionally, there was up-regulation of *FOXO3* and *FOXO4* following the combination therapy (Supplementary Information 1). We identified several known *FOXO* target genes, such as *CDKN1A*, *CDKN2A*, *MYC*, *TXNRD1*, *GPX*, *CACNA1C*, *APOC1*, *DUSP5*, *PIK3C*, *HER2*, and *HER3*, to be up-regulated or maintained and this is consistent with possible transcriptional activation of FOXO functions. This observation was supported by the significant up-regulation or maintenance of the expression of JNK, DUSP4-6 with significant differential expression of several isoforms of 14-3-3 protein, which are known positive regulators of FOXO transcriptional activity [69,70,71,72,73]. It is conceivable that increased JNK expression and function perturbs the capacity of high pAKT levels to inhibit FOXO function [74,75,76]. An increased FOXO function promotes cellular reprogramming, cell cycle arrest and apoptosis [77,78,79], and the two latter processes have, in particular, appeared to be more pronounced in combination therapy [28].

A recent study [80] revealed the cause of the refractory nature of thyroid and colorectal cancer cells towards MAPK inhibition and uncovered similar emergence of compensatory signalling involving HER3 following drug treatment. It has been found that ERK is involved in HER3 transcriptional regulation by modulating the activities of C-terminal binding protein 1 and 2 (CTBP-1,2), a transcriptional repressor of HER3 gene.

Our joint analysis of gene and phospho-protein expression following therapy suggests the involvement of pERK in HER3 transcriptional regulation. The significant up-regulation of HER3 was observed after strong inhibition of pERK but not pAKT by combination treatment of SKOV3 tumour xenograft (Figure 10A and B). This may mean that pERK phosphorylates FOXO as pAKT and pERK inhibition can induce FOXO trafficking to the nucleus and up-regulation of HER3. The involvement of pERK together with pAKT in FOXO phosphorylation was observed in PI3K/AKT and MEK/ERK pathway inhibition experiments where combination inhibition acts synergistically to enhance activation of FOXO transcription factors [81].

Garrett *et al.* [18] studied the effects of lapatinib, an EGFR and HER2 dual kinase inhibitor [82], and found that a panel of breast cancer cell lines with HER2 overexpression responded to it by exhibiting transcriptional and post-translational up-regulation of HER3. Further dissection into the biochemical pathway revealed its mechanism that involved relief of negative regulation of FOXO3a transcription factor exerted by PI3K/AKT pathway following lapatinib treatment. Hence, lapatinib caused inhibition of the EGFR/HER2/PI3K/AKT pathway that led to nuclear abundance of FOXO3a, which in turn caused up-regulation of *HER3* gene.

Key questions arising are whether an increase in pERK as a function of increasing HER3 abundance is specifically HER3-dependent and whether this would lead to a re-adjustment in growth signalling, even in combination treatment. Recently, it was reported that a combination treatment targeting HER2 might not be effective in completely eliminating growth signals owing to HER3 up-regulation [83]. Trastuzumab or lapatinib led to induction of HER3 expression, which only caused a partial repression of pAKT and pERK levels that were still enough to promote tumour growth. However, co-treatment of HER3 specific monoclonal antibody U3-1287 resulted in complete repression of pERK leading to better survival of mice bearing HER2 overexpressing xenografts than the dual combination treatment alone [47]. This addresses both of the above questions. The argument that drug induced HER3 up-regulation, even during combination treatment, would counteract the sensitivity process is supported by a recent study [83].

Taken together, these experimental and clinical data show stringent regulation of ErbB co-expression through multiple feedback loops at the genetic level. Drugs targeting receptors and inhibiting PI3K/AKT and ERK1/2 signalling activate these feedbacks and this activation leads to the abrogation of drug effects. An account of emerging genetic feedbacks and their regulation of ErbB composition must then become an integral part of the monitoring of drug effectiveness during prolonged treatment of cancer.

The systems approach proposed in this paper considers these feedback loops phenomenologically through the incorporation of gene expression data into the kinetic model. To consider this effect more fully the model should be developed further to include the mechanisms of genetic feedback control of receptor co-expression and close signalling activation loops, and our work [58] provides the very beginnings of this development. Through the extended modelling framework it would be possible to then analyse the involvement of different outcomes of ErbB signalling, particularly pAKT and pERK, in those regulatory feedbacks. This extended model can be used as a tool to investigate correlations between cellular signal inhibition and tumour growth over a long time period of drug administration; it is over these long periods of time when signalling network reprogramming becomes a critical influence on drug effectiveness. Development of this systems approach requires a new strategy for the collection of experimental data. Dynamic proteomic and genomic data become necessary, offering more than the static data sets used here. The possibility to track temporal activation of gene expression controlling RTK co-expression following drug intervention will allow modelling and monitoring the temporal response of cancer cells to long-term drug therapy.

## 4. Conclusion

As a result of recent experimental and clinical research on the dynamic activity of RTK networks, novel insights were gained on gene–gene interaction networks underlying the genetic reprogramming of RTK during tumour growth and RTK-targeted drug therapy. Assessment of the impact of RTK reprogramming on drug resistance and synergy during prolonged therapy can inform the rational development of effective strategies of drug therapy, particularly combination therapy, and help to design biomarkers for anti-RTK treatment. Computational systems approaches incorporating dynamic change in gene expression profile provide support for experimental and clinical trial design and interpretation of combination therapy. Here, we applied a computational systems approach to investigate trastuzumab and pertuzumab mono- and combination therapies depending on dynamically changing HER2 and HER3 receptor composition and showed that the effectiveness of these three therapies depends on HER3/HER2 dimerization kinetics which is under the strict control of HER3/HER2 co-expression. We showed that, in contrast to mono-therapy, which is receptor composition dependent, combination therapy shows composition-independent inhibition effect in a wide range of HER3/HER2 composition. This feature of the drug combination can contribute significantly to its synergistic action on cellular signalling and tumour growth at the dynamically changing receptor composition and suppression of cancer cell addiction to compensatory control program in HER2 overexpressing tumours which commonly leads to resistance to single therapy.

## Supplementary Files

Supplementary File 1 Supplementary (XLS, 152 KB)

Supplementary File 2 Supplementary 2 (PDF, 179 KB)

Supplementary File 3 Supplementary 3 (PDF, 60 KB)

## Supplementary Materials

Supplementary materials can be accessed at: http://www.mdpi.com/2073-4409/3/2/563/s1, http://www.mdpi.com/2073-4409/3/2/563/s2 and http://www.mdpi.com/2073-4409/3/2/563/s3.
