# Supplementary material for: Systems Analysis of Drug-Induced Receptor Tyrosine Kinase Reprogramming Following Targeted Mono- and Combination Anti-Cancer Therapy
Source: Cells. 2014 Jun 10;3(2):563–91. doi: 10.3390/cells3020563 (PMC4092865; doi:10.3390/cells3020563)

Supplementary Material 2

Figure 1. Network illustration of the ERBB signaling pathway with Trastuzumab treatment. The color of the node depicts the ratio of the expression values with drug compared to the expression values in control samples.

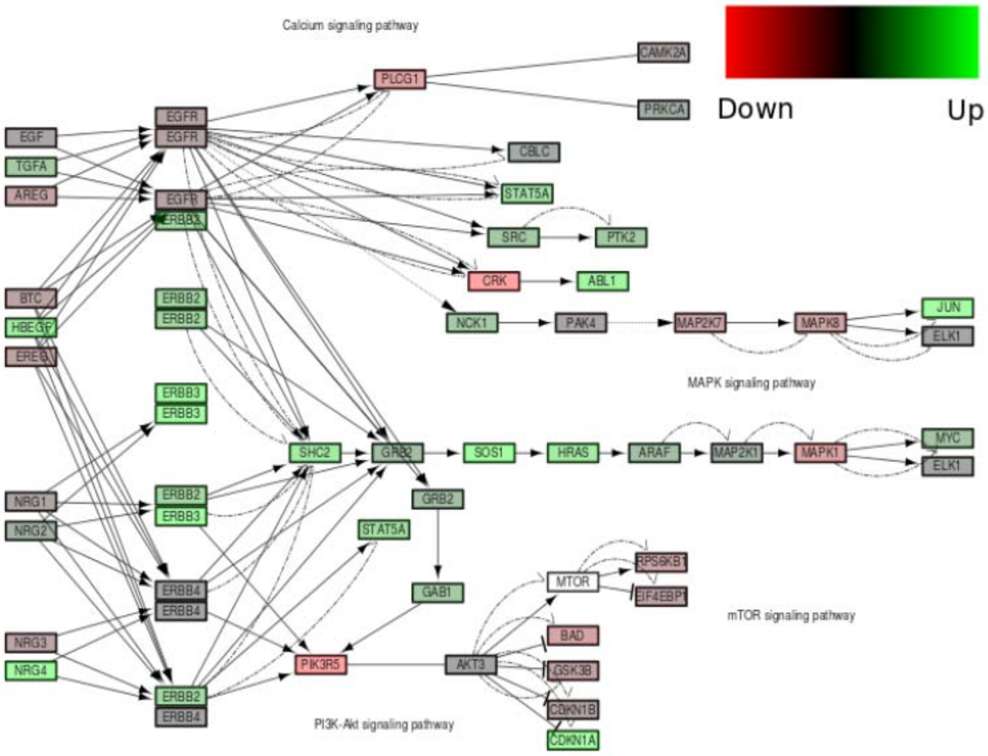

Figure 2. Network illustration of the ERBB signaling pathway with Pertuzumab treatment. The color of the node depicts the ratio of the expression values with drug compared to the expression values in control samples.

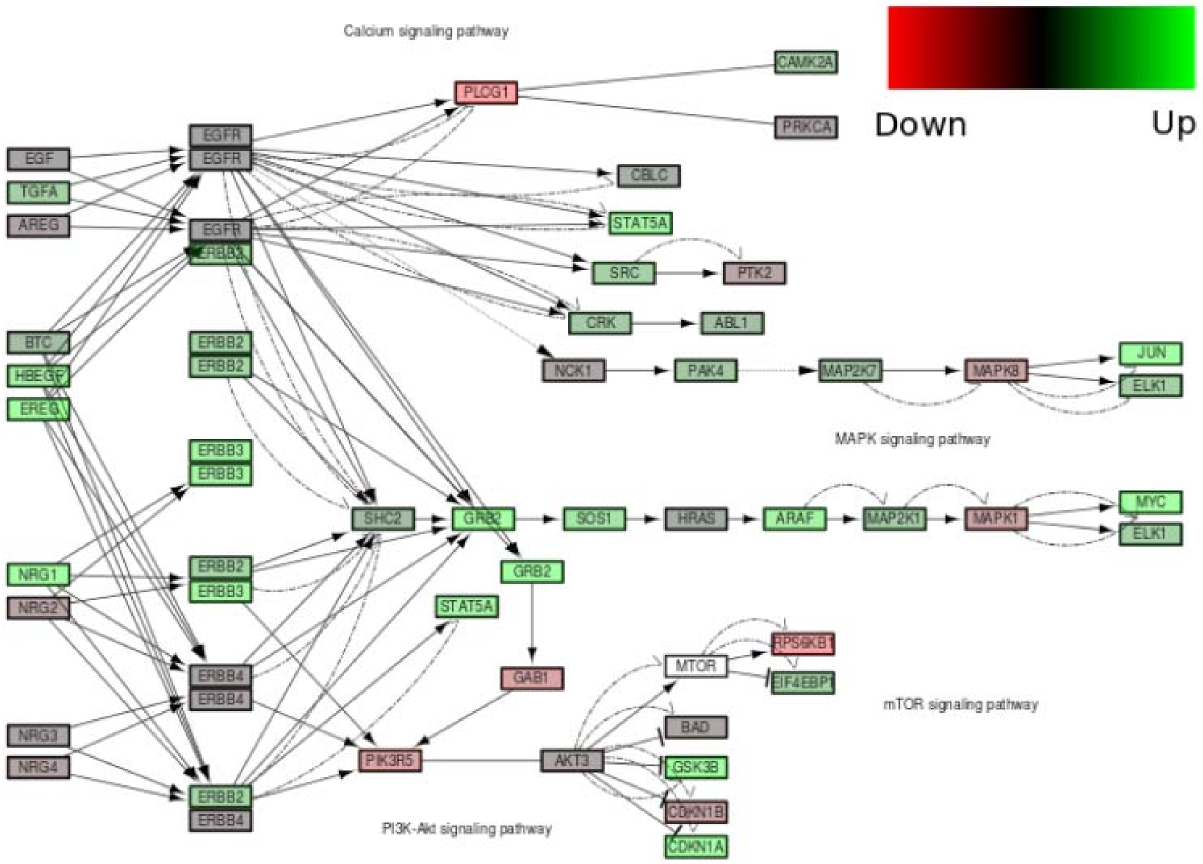

Figure 3. Network illustration of the ERBB signaling pathway with Combination treatment. The color of the node depicts the ratio of the expression values with drug compared to the expression values in control samples.

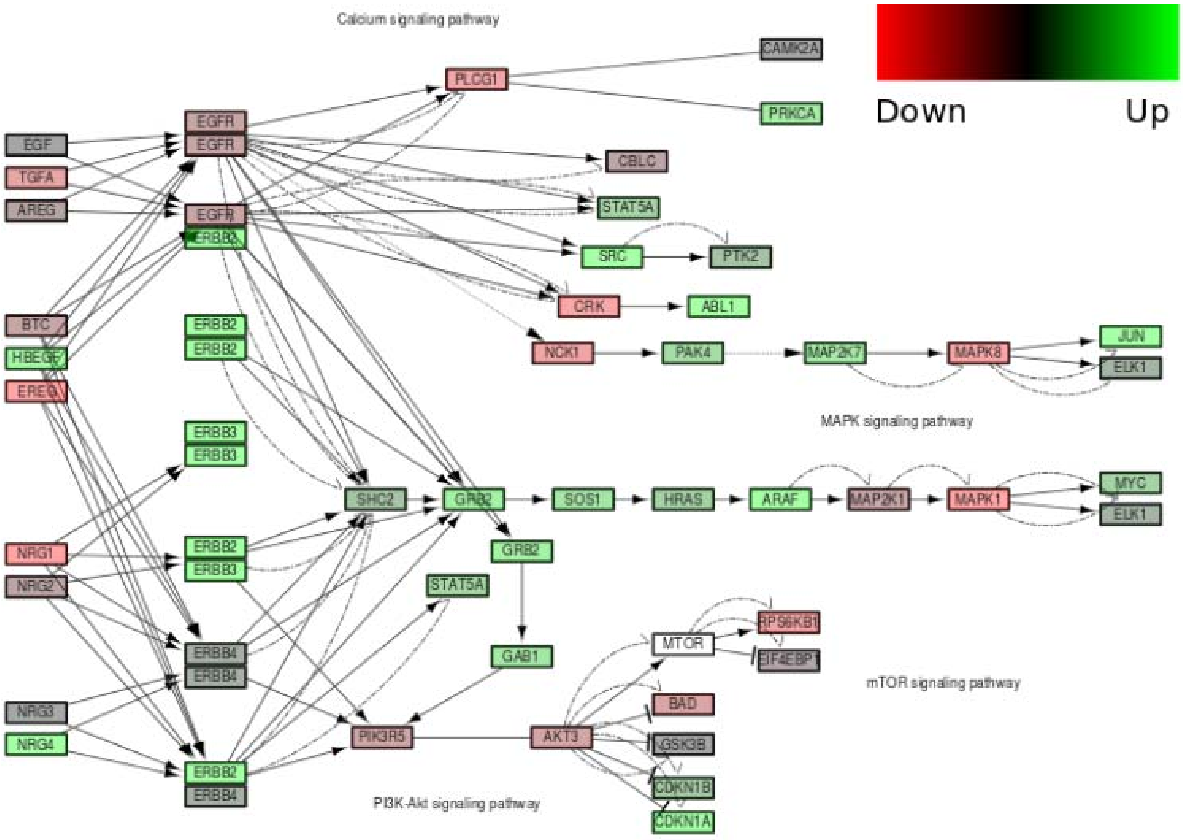

Supplement: Supplementary File 2 — Supplementary 2 (PDF, 179 KB) [file cells-03-00563-s002.pdf]
