# Supplementary material for: Systems Analysis of Drug-Induced Receptor Tyrosine Kinase Reprogramming Following Targeted Mono- and Combination Anti-Cancer Therapy
Source: Cells. 2014 Jun 10;3(2):563–91. doi: 10.3390/cells3020563 (PMC4092865; doi:10.3390/cells3020563)
Supplement: Supplementary File 3 — Supplementary 3 (PDF, 60 KB) [file cells-03-00563-s003.pdf]

Supplementary 3

Mathematical model of Ras/RAF/MEK/ERK and PI3K/PTEN/AKT pathway

System of ordinary differential equations

$$\frac{d[\text{E3}]}{dt} = -\text{V}_1 \quad \text{(S3.1)}$$

$$\frac{d[\text{E3H}]}{dt} = \text{V}_1 - \text{V}_2 - \text{V}_{51} - \text{V}_{66} \quad \text{(S3.2)}$$

$$\frac{d[\text{HRG}]}{dt} = -\text{V}_1 \quad \text{(S3.3)}$$

$$\frac{\text{d}[\text{E3H}_\text{c}]}{\text{dt}} = \text{V}_{51} - \text{V}_{52} \quad \text{(S3.4)}$$

$$\frac{d[\text{E2}]}{dt} = -\text{V}_2 - \text{V}_{49} - \text{V}_{52} - \text{V}_{59} - \text{V}_{65} - \text{V}_{72} - \text{V}_{85} \quad \text{(S3.5)}$$

$$\frac{d[\text{E23H}]}{dt} = \text{V}_2 - \text{V}_3 + \text{V}_4 + \text{V}_{52} - \text{V}_{53} \quad \text{(S3.6)}$$

$$\frac{d[\text{E23H}_\text{c}]}{dt} = \text{V}_{53} - \text{V}_{54} \quad \text{(S3.7)}$$

$$\frac{d[\text{pE23H}]}{dt} = \text{V}_3 - \text{V}_4 - \text{V}_5 + \text{V}_8 - \text{V}_{27} + \text{V}_{29} - \text{V}_{48} + \text{V}_{54} \quad \text{(S3.8)}$$

$$\frac{d[\text{Shc}]}{dt} = -\text{V}_5 + \text{V}_{10} - \text{V}_{60} \quad \text{(S3.9)}$$

$$\frac{d[\text{pE23H-Shc}]}{dt} = \text{V}_5 - \text{V}_6 \quad \text{(S3.10)}$$

$$\frac{d[\text{pE23H-pShc}]}{dt} = \text{V}_6 - \text{V}_7 \quad \text{(S3.11)}$$

$$\frac{d[\text{GS}]}{dt} = -\text{V}_7 + \text{V}_9 - \text{V}_{63} - \text{V}_{71} \quad \text{(S3.12)}$$

$$\frac{d[\text{Shc-GS}]}{dt} = \text{V}_8 - \text{V}_9 - \text{V}_{64} - \text{V}_{71} - \text{V}_{77} - \text{V}_{83} \quad \text{(S3.13)}$$

$$\frac{d[\text{pShc}]}{dt} = \text{V}_9 - \text{V}_{10} \quad \text{(S3.14)}$$

$$\frac{d[\text{pE23H-pShc-GS}]}{dt} = \text{V}_7 - \text{V}_8 \quad \text{(S3.15)}$$

$$\frac{d[\text{ppAkt-PIP3}]}{dt} = \text{V}_{44} - \text{V}_{45} \quad \text{(S3.16)}$$

$$\frac{d[\text{Ras-GDP}]}{dt} = -\text{V}_{11} + \text{V}_{12} \quad \text{(S3.17)}$$

$$\frac{d[\text{Ras-GTP}]}{dt} = \text{V}_{11} - \text{V}_{12} \quad \text{(S3.18)}$$

$$\frac{d[\text{Raf}]}{dt} = -\text{V}_{13} + \text{V}_{14} \quad \text{(S3.19)}$$

$$\frac{d[\text{Raf*}]}{dt} = \text{V}_{13}\text{-V}_{14} \quad (S3.20)$$

$$\frac{d[\text{MEK}]}{dt} = \text{-V}_{15}+\text{V}_{18} \quad (S3.21)$$

$$\frac{d[\text{pMEK}]}{dt} = \text{V}_{15}\text{-V}_{16}\text{-V}_{19}+\text{V}_{22} \quad (S3.22)$$

$$\frac{d[\text{PP2A}]}{dt} = \text{-V}_{16}+\text{V}_{18}\text{-V}_{20}+\text{V}_{22}\text{-V}_{41}+\text{V}_{43}\text{-V}_{45}+\text{V}_{47} \quad (S3.23)$$

$$\frac{d[\text{MEKP-PP2A}]}{dt} = \text{V}_{16}\text{-V}_{17}+\text{V}_{21}\text{-V}_{22} \quad (S3.24)$$

$$\frac{d[\text{MEK-PP2A}]}{dt} = \text{V}_{17}\text{-V}_{18} \quad (S3.25)$$

$$\frac{d[\text{pAkt-PIP3-PP2A}]}{dt} = \text{V}_{41}\text{-V}_{42}+\text{V}_{46}\text{-V}_{47} \quad (S3.26)$$

$$\frac{d[\text{ppMEKPP-PP2A}]}{dt} = \text{V}_{20}\text{-V}_{21} \quad (S3.27)$$

$$\frac{d[\text{ppMEKPP-PP2A}]}{dt} = \text{V}_{20}\text{-V}_{21} \quad (S3.28)$$

$$\frac{d[\text{ppMEK}]}{dt} = \text{V}_{19}\text{-V}_{20} \quad (S3.29)$$

$$\frac{d[\text{ERK}]}{dt} = \text{-V}_{23}+\text{V}_{24} \quad (S3.30)$$

$$\frac{d[\text{pERKP}]}{dt} = \text{V}_{23}\text{-V}_{24}\text{-V}_{25}+\text{V}_{26} \quad (S3.31)$$

$$\frac{d[\text{ppERK}]}{dt} = \text{V}_{25}\text{-V}_{26} \quad (S3.32)$$

$$\frac{d[\text{ppAkt-PIP3-PP2A}]}{dt} = \text{V}_{45}\text{-V}_{46} \quad (S3.33)$$

$$\frac{d[\text{Akt-PIP3}]}{dt} = \text{V}_{39}\text{-V}_{40}+\text{V}_{43} \quad (S3.34)$$

$$\frac{d[\text{PI3K}]}{dt} = -\text{V}_{27}+\text{V}_{30}-\text{V}_{58}-\text{V}_{84} \quad (S3.35)$$

$$\frac{d[\text{pE23H-PI3K}]}{dt} = \text{V}_{27} \quad (S3.36)$$

$$\frac{d[\text{PI}]}{dt} = \text{-V}_{31}+\text{V}_{34} \quad (S3.37)$$

$$\frac{d[\text{PTEN}]}{dt} = -\text{V}_{32}+\text{V}_{34}-\text{V}_{35}-\text{V}_{36}+2\text{V}_{38} \quad (S3.38)$$

$$\frac{d[\text{PIP3}]}{dt} = \text{-V}_{32}\text{-V}_{39} \quad (S3.39)$$

$$\frac{d[\text{PTEN-PIP3}]}{dt} = \text{V}_{32}\text{-V}_{33} \quad (S3.40)$$

$$\frac{d[\text{PTEN-PI}]}{dt} = \text{V}_{33}\text{-V}_{34} \quad (S3.41)$$

$$\frac{d[\mathrm{pPTEN}]}{dt} = \mathrm{V}_{35}\text{-}\mathrm{V}_{36} \quad (S3.42)$$

$$\frac{d[\mathrm{pPTENP\text{-}PTEN}]}{dt} = \mathrm{V}_{36}\text{-}\mathrm{V}_{37} \quad (S3.43)$$

$$\frac{d[\mathrm{PTEN\text{-}PTEN}]}{dt} = \mathrm{V}_{37}\text{-}\mathrm{V}_{38} \quad (S3.44)$$

$$\frac{d[\mathrm{PI3K^{*}\text{-}PI}]}{dt} = \mathrm{V}_{31}\text{-}\mathrm{V}_{55} \quad (S3.45)$$

$$\frac{d[\mathrm{PI3K^{*}\text{-}PIP3}]}{dt} = \mathrm{V}_{55}\text{-}\mathrm{V}_{56} \quad (S3.46)$$

$$\frac{d[\mathrm{Akt}]}{dt} = \text{-}\mathrm{V}_{39} \quad (S3.47)$$

$$\frac{d[\mathrm{pAkt\text{-}PIP3}]}{dt} = \mathrm{V}_{40}\text{-}\mathrm{V}_{41}\text{-}\mathrm{V}_{44}+\mathrm{V}_{47} \quad (S3.48)$$

$$\frac{d[\mathrm{Akt\text{-}PIP3\text{-}PP2A}]}{dt} = \mathrm{V}_{42}\text{-}\mathrm{V}_{43} \quad (S3.49)$$

$$\frac{d[2C4]}{dt} = \text{-}\mathrm{V}_{49} \quad (S3.50)$$

$$\frac{d[\mathrm{E2\text{-}2C4}]}{dt} = \mathrm{V}_{49}\text{-}\mathrm{V}_{50} \quad (S3.51)$$

$$\frac{d[\mathrm{E2\text{-}2C4_c}]}{dt} = \mathrm{V}_{50} \quad (S3.52)$$

$$\frac{d[\mathrm{E22}]}{dt} = \mathrm{V}_{59} - \mathrm{V}_{60} \quad (S3.53)$$

$$\frac{d[p\mathrm{E22}]}{dt} = \mathrm{V}_{60} \quad (S3.54)$$

$$\frac{d[p\mathrm{E22\_Sch}]}{dt} = \mathrm{V}_{61} \quad (S3.55)$$

$$\frac{d[p\mathrm{E22\text{-}pSch}]}{dt} = \mathrm{V}_{62} \quad (S3.56)$$

$$\frac{d[p\mathrm{E22\text{-}pSch\text{-}GS}]}{dt} = \mathrm{V}_{63} - \mathrm{V}_{64} \quad (S3.57)$$

$$\frac{d[\mathrm{E2\text{-}Tr}]}{dt} = \mathrm{V}_{65} \quad (S3.58)$$

$$\frac{d[\mathrm{E23H\text{-}Tr}]}{dt} = \mathrm{V}_{66} \quad (S3.59)$$

$$\frac{d[p\mathrm{E23H\text{-}Tr}]}{dt} = \mathrm{V}_{67} \quad (S3.60)$$

$$\frac{d[p\mathrm{E23H\text{-}Tr\text{-}Shc}]}{dt} = \mathrm{V}_{68} \quad (S3.61)$$

$$\frac{d[p\mathrm{E23H\text{-}Tr\text{-}pShc}]}{dt} = \mathrm{V}_{69} \quad (S3.62)$$

$$\frac{d[p\mathrm{E23H\text{-}Tr\text{-}pShc\text{-}GS}]}{dt} = \mathrm{V}_{70} - \mathrm{V}_{71} \quad (S3.63)$$

$$\frac{d[\text{E22-Per}]}{dt} = \text{V}_{72} \tag{S3.64}$$

$$\frac{d[p\text{E22-Per}]}{dt} = \text{V}_{73} \tag{S3.65}$$

$$\frac{d[p\text{E22-Sch-Per}]}{dt} = \text{V}_{74} \tag{S3.66}$$

$$\frac{d[p\text{E22-Per-pSch}]}{dt} = \text{V}_{75} \tag{S3.67}$$

$$\frac{d[p\text{E22-Per-pSch-GS}]}{dt} = \text{V}_{76} - \text{V}_{77} \tag{S3.68}$$

$$\frac{d[\text{E22-Per2}]}{dt} = \text{V}_{78} \tag{S3.69}$$

$$\frac{d[p\text{E22-Per2}]}{dt} = \text{V}_{79} \tag{S3.70}$$

$$\frac{d[p\text{E22-Per2-Shc}]}{dt} = \text{V}_{80} \tag{S3.71}$$

$$\frac{d[p\text{E22-Per2-pShc}]}{dt} = \text{V}_{81} \tag{S3.72}$$

$$\frac{d[p\text{E22-Per2-pShc-GS}]}{dt} = \text{V}_{82} - \text{V}_{83} \tag{S3.73}$$

$$\frac{d[p\text{E23H-Tr-PI3K}]}{dt} = \text{V}_{84} \tag{S3.74}$$

$$\frac{d[\text{E2-Per}]}{dt} = \text{V}_{85} \tag{S3.75}$$

$$\frac{d[\text{E2-Tr-Per}]}{dt} = \text{V}_{86}\,, \tag{S3.76}$$

where reaction rates V_i are determined by the following equations:

$$\text{V}_1 = \text{k}_1 \cdot ([\text{E3}] \cdot [\text{HRG}] - \text{K}_{\text{d},1} \cdot [\text{E3H}]) \tag{S3.77}$$

$$\text{V}_2 = \text{k}_2 \cdot ([\text{E3H}] \cdot [\text{E2}] - \text{K}_{\text{d},2} \cdot [\text{E23H}]) \tag{S3.78}$$

$$\text{V}_3 = \text{k}_3 \cdot [\text{E23H}]/(\text{K}_{\text{d},3} + [\text{E23H}]) \tag{S3.79}$$

$$\text{V}_4 = \text{V}_{\text{max},4} \cdot [\text{pE23H}] \;/\; (\text{K}_{\text{m},4} + [\text{pE23H}]) \tag{S3.80}$$

$$\text{V}_5 = \text{k}_5 \cdot ([\text{pE23H}] \cdot [\text{Shc}] - \text{K}_{\text{d},5} \cdot [\text{pE23H-Shc}]) \tag{S3.81}$$

$$\text{V}_6 = \text{k}_6 \cdot [\text{pE23H-Shc}]/(\text{K}_{\text{d},6} + [\text{pE23H-Shc}]) \tag{S3.82}$$

$$\text{V}_7 = \text{k}_7 \cdot ([\text{pE23H-pShc}] \cdot [\text{GS}] - \text{K}_{\text{d},7} \cdot [\text{pE23H-pShc-GS}]) \tag{S3.83}$$

$$\text{V}_8 = \text{k}_8 \cdot ([\text{pE23H-Shc-GS}] - \text{K}_{\text{d},8} \cdot [\text{pE23HP}] \cdot [\text{Shc-GS}]) \tag{S3.84}$$

$$V_9 = k_9 \cdot [Shc\text{-}GS] - k_{-9} \cdot [pShc] \cdot [GS] \quad (S3.85)$$

$$V_{10} = V_{max,10} \cdot [pShc] / (K_{m,10} + [pShc]) \quad (S3.86)$$

$$V_{11} = k_{11} \cdot [Ras\text{-}GDP] \cdot ([Shc\text{-}GS] + [pE23H\text{-}Shc\text{-}GS] + [pE22\text{-}pShc\text{-}GS] + \\ + [pE23H\text{-}Tr\text{-}pShc\text{-}GS] + [pE22\text{-}Per\text{-}pShc\text{-}GS] + [pE22\text{-}Per2\text{-}pShc\text{-}GS])? \\ (K_{m,11} + [Ras\text{-}GDP]) \quad (S3.87)$$

$$V_{12} = V_{12} \cdot [Ras\text{-}GTP]/(K_{m,12} + [Ras\text{-}GTP]) \quad (S3.88)$$

$$V_{13} = k_{13} \cdot [Raf] \cdot [Ras\text{-}GTP]/(K_{m,13} + [Raf]) \quad (S3.89)$$

$$V_{14} = k_{14} \cdot [Raf^*] \cdot ([ppAkt\text{-}PIP3] + [E_{Raf}])/([Raf^*] + K_{m,14}) \quad (S3.90)$$

$$V_{15} = k_{15} \cdot [MEK] \cdot [Raf^*]/(K_{m,15} + [MEK]) \quad (S3.91)$$

$$V_{16} = k_{16} \cdot [pMEK] \cdot [PP2A] \quad (S3.92)$$

$$V_{17} = k_{cat,16} \cdot [pMEK\text{-}PP2A] \quad (S3.93)$$

$$V_{18} = k_{18} \cdot [MEK\text{-}PP2A] \quad (S3.94)$$

$$V_{19} = k_{15} \cdot [pMEK] \cdot [Raf^*]/(K_{m,15} + [pMEK]) \quad (S3.95)$$

$$V_{20} = k_{16} \cdot ([PP2A] \cdot [ppMEK] - K_{d,16} \cdot [ppMEK\text{-}PP2A]) \quad (S3.96)$$

$$V_{21} = k_{cat,16} \cdot [ppMEK\text{-}PP2A] \quad (S3.97)$$

$$V_{22} = k_{22} \cdot [pMEK\text{-}PP2A] \quad (S3.98)$$

$$V_{23} = k_{23} \cdot [ERK] \cdot [ppMEK]/(K_{m,23} + [ERK]) \quad (S3.99)$$

$$V_{24} = V_{max,24} \cdot [pERK]/(K_{m,24} + [pERK]) \quad (S3.80)$$

$$V_{25} = k_{23} \cdot [ppMEK] \cdot [pERK]/(K_{m,23} + [pERK]) \quad (S3.81)$$

$$V_{26} = V_{max,24} \cdot [ppERK]/(K_{m,24} + [ppERK]) \quad (S3.82)$$

$$V_{27} = k_{27} \cdot ([pE23H] \cdot [PI3K] - K_{d,27} \cdot [pE23H\text{-}PI3K]) \quad (S3.83)$$

$$V_{28} = k_{28} \cdot [pE23H\text{-}PI3K] \quad (S3.84)$$

$$V_{29} = k_{29} \cdot [\text{pE23H-PI3K*}] \quad \text{(S3.85)}$$

$$V_{30} = k_{30} \cdot [\text{pE23H-PI3K}] \quad \text{(S3.86)}$$

$$V_{31} = k_{31} \cdot [\text{PI}] \cdot ([\text{pE23H-PI3K}] + [\text{pE23H-Tr-PI3K}])/(K_{d,31} + [\text{PI}]) \quad \text{(S3.87)}$$

$$V_{32} = k_{32} \cdot ([\text{PIP3}] \cdot [\text{PTEN}] - K_{d,32} \cdot [\text{PTEN-PIP3}]) \quad \text{(S3.88)}$$

$$V_{33} = k_{33} \cdot [\text{PTEN-PIP3}] \quad \text{(S3.89)}$$

$$V_{34} = k_{34} \cdot [\text{PTEN-PI}] \quad \text{(S3.90)}$$

$$V_{35} = V_{max,35} \cdot [\text{PTEN}]/(K_{m,35} + [\text{PTEN}]) \quad \text{(S3.91)}$$

$$V_{36} = k_{36} \cdot ([\text{PTEN}] \cdot [\text{pPTEN}] - K_{d,36} \cdot [\text{pTENP-PTEN}]) \quad \text{(S3.92)}$$

$$V_{37} = k_{cat,37} \cdot [\text{pPTENP-PTEN}] \quad \text{(S3.93)}$$

$$V_{38} = k_{38} \cdot [\text{PTEN-PTEN}] \quad \text{(S3.94)}$$

$$V_{39} = k_{39} \cdot ([\text{PIP3}] \cdot [\text{Akt}] - K_{d,39} \cdot [\text{Akt-PIP3}]) \quad \text{(S3.95)}$$

$$V_{40} = V_{max,40} \cdot [\text{Akt-PIP3}]/(K_{m,40} + [\text{Akt-PIP3}]) \quad \text{(S3.96)}$$

$$V_{41} = k_{41} \cdot ([\text{pAkt-PIP3}] \cdot [\text{PP2A}] - K_{d,41} \cdot [\text{ppAkt-PIP3-PP2A}]) \quad \text{(S3.97)}$$

$$V_{42} = k_{cat,42} \cdot [\text{pAkt-PIP3-PP2A}] \quad \text{(S3.98)}$$

$$V_{43} = k_{43} \cdot [\text{Akt-PIP3-PP2A}] \quad \text{(S3.99)}$$

$$V_{44} = V_{max,40} \cdot [\text{pAkt-PIP3}]/(K_{m,40} + [\text{pAkt-PIP3}]) \quad \text{(S3.100)}$$

$$V_{45} = k_{45} \cdot ([\text{ppAkt-PIP3}] \cdot [\text{PP2A}] - K_{d,45} \cdot [\text{ppAkt-PIP3-PP2A}]) \quad \text{(S3.101)}$$

$$V_{46} = k_{cat,46} \cdot [\text{ppAkt-PIP3-PP2A}] \quad \text{(S3.102)}$$

$$V_{47} = k_{47} \cdot [\text{pAkt-PIP3-PP2A}] \quad \text{(S3.103)}$$

$$V_{48} = k_{48} \cdot [\text{pE23H}] \quad \text{(S3.104)}$$

$$V_{49} = k_{49} \cdot ([\text{2C4}] \cdot [\text{E2}] - K_{d,49} \cdot [\text{E2-2C4}]) \quad \text{(S3.105)}$$

$$V_{50} = k_{50} \cdot [\text{E2-2C4}] - k_{-50} \cdot [\text{E2-2C4}_c] \quad \text{(S3.106)}$$

$V_{51} = k_{51} \cdot [E3H]$ (S3.107)

$V_{52} = k_2 \cdot ([E3H_c] \cdot [E2] - K_{d,2} \cdot [E23H])$ (S3.108)

$V_{53} = k_{53} \cdot [E23H]$ (S3.109)

$V_{54} = k_3 \cdot ([E23H_c] - K_{d,3} \cdot [pE23H])$ (S3.110)

$V_{55} = k_{55} \cdot [PI3K\text{-}PI]$ (S3.111)

$V_{56} = k_{56} \cdot [PI3K\text{-}PIP3]$ (S3.112)

$V_{57} = k_{57} \cdot ([PTEN] \cdot [bpV] - K_{d,57} \cdot [PTEN\_bpV])$ (S1.113)

$V_{58} = k_{58} \cdot ([PI3K] \cdot [LY] - K_{d,58} \cdot [PI3K\_LY])$ (S3.114)

$V_{59} = k_{59} \cdot ([E2] \cdot [E2] - K_{d,59} \cdot [E22])$ (S3.115)

$V_{60} = k_3 \cdot [E22]/(K_{d,3} + [E22]) - V_{max,4} [pE22]/(K_{m,4} + [pE22])$ (S3.116)

$V_{61} = k_5 \cdot (2 \cdot [pE22] \cdot [Shc] - K_{d,5} \cdot [pE22\text{-}Shc])$ (S3.117)

$V_{62} = k_6 \cdot [pE22\text{-}Shc])/(K_{d,6} + [pE22\text{-}Shc])$ (S3.118)

$V_{63} = k_7 \cdot ([pE22\text{-}pShc][GS] - K_{d,7}[pE22\text{-}pShc\text{-}GS])$ (S3.119)

$V_{64} = k_8 \cdot ([pE22\text{-}pShc\text{-}GS] - K_{d,8}[pE22\text{-}Per][Shc\text{-}GS])$ (S3.120)

$V_{65} = k_{60} \cdot ([Tr] \cdot [E2] - K_{d,60} \cdot [E2\text{-}Tr])$ (S3.121)

$V_{66} = k_2 \cdot ([E3H] \cdot [E2\text{-}Tr] - K_{d,2} \cdot [E23H\text{-}Tr])$ (S3.122)

$V_{67} = k_3 \cdot [E23\text{-}Tr]/(K_{d,3} + [E23\text{-}Tr]) - V_{max,4} [pE23\text{-}Tr]/(K_{m,4} + [pE23\text{-}Tr])$ (S3.123)

$V_{68} = k_5 \cdot ([pE23H\text{-}Tr] \cdot [Shc] - K_{d,5} \cdot [pE23H\text{-}Tr\text{-}Shc])$ (S3.124)

$V_{69} = k_6 \cdot [pE23H\text{-}Tr\text{-}Shc])/(K_{d,6} + pE23H\text{-}Tr\text{-}Shc)$ (S3.125)

$V_{70} = k_7 \cdot ([pE23H\text{-}pShc\text{-}Tr] \cdot [GS] - K_{d,7} \cdot [pE23H\text{-}Tr\text{-}pShc\text{-}GS])$ (S3.126)

$V_{71} = k_8 \cdot ([pE23H\text{-}Tr\text{-}pShc\text{-}GS] - K_{d,8}[pE23H\text{-}Tr][Shc\text{-}GS])$ (S3.127)

$V_{72} = k_{59} \cdot ([E2] \cdot [E2\text{-}Per] - K_{d,59} \cdot [E22\text{-}Per])$ (S3.128)

$V_{73} = k_3 \cdot [E22\text{-}Per]/(Kd,3 + [E22\text{-}Per]) - V_{max,4} [pE22\text{-}Per]/(K_{m,4} + [pE22\text{-}Per])$ (S3.129)

$V_{74} = k_5 \cdot (2\ [pE22\text{-}Per] \cdot [Shc] - K_{d,5} \cdot [pE22\text{-}Per\text{-}Shc])$ (S3.130)

$V_{75} = k_6 \cdot [pE22\text{-}Per\text{-}Shc])/(K_{d,6} + [pE22\text{-}Per\text{-}Shc])$ (S3.131)

$V_{76} = k_7 \cdot ([pE22\text{-}Per\text{-}pShc][GS] - K_{d,7}[pE22\ \text{-}Per\text{-}pShc\text{-}GS])$ (S3.132)

$V_{77} = k_8 \cdot ([pE22\text{-}Per\text{-}pShc\text{-}GS] - K_{d,8}[pE22\text{-}Per][Shc\text{-}GS])$ (S3.133)

$V_{78} = k_{59} \cdot ([E2\text{-}Per] \cdot [E2\_Per] - K_{d,59} \cdot [E22\text{-}Per2])$ (S3.134)

$V_{79} = k_3 \cdot [E22\text{-}Per2]/(K_{d,3} + [E22\text{-}Per2]) -- V_{max,4} [pE22\text{-}Per2]/(K_{m,4} + [pE22\text{-}Per2])$ (S3.135)

$V_{80} = k_5 \cdot (2 \cdot [pE22\text{-}Per2] \cdot [Shc] - K_{d,5} \cdot [pE22\text{-}Per2\text{-}Shc])$ (S3.136)

$V_{81} = k_6 \cdot [pE22\text{-}Per\text{-}Shc])/(K_{d,6} + pE22\text{-}Per\text{-}Shc)$ (S3.137)

$V_{82} = k_7 \cdot ([pE22\ \text{-}Per2\text{-}pShc][GS] - K_{d,7}[pE22\ \text{-}Per2\text{-}pShc\ \text{-}GS]\ )$ (S3.138)

$V_{83} = k_8 \cdot ([pE22\text{-}Per2\text{-}pShc\text{-}GS] - K_{d,8}[pE22\text{-}Per2][Shc\text{-}GS])$ (S3.139)

$V_{84} = k_{27} \cdot ([pE23H\text{-}Tr] \cdot [PI3K] - K_{d,27} \cdot [pE23H\text{-}Tr\text{-}PI3K])$ (S3.140)

$V_{85} = k_5 \cdot ([E2] \cdot [Per] - K_{d,5} \cdot [E2\text{-}Per])$ (S3.141)

$V_{86} = k_5 \cdot ([E2\text{-}Tr] \cdot [Per] - K_{d,5} \cdot [E2\text{-}Tr\text{-}Per])$ (S3.142)

Table 1. Abbreviations used in the model

Abbreviations in ODEs	Protein names
E2	ErbB2 (HER2) receptor
E3	ErbB3 (HER3) receptor
HRG	Heregulin
E3H	ErbB3/HRG ligand/receptor complex
$E3H_c$	ErbB3/HRG ligand/receptor complex
E23H	Heterodimer of ErbB3/HRG with ErbB2
$E23H_c$	Heterodimer of ErbB3/HRG with ErbB2
pE23H, pHER2	Phosphorylated heterodimer of ErbB3/HRG with ErbB2
Grb2	growth factor receptor-binding protein 2
Ras-GDP	Ras-GDP protein
Ras-GTP	Ras-GTP protein
Raf	Raf protein
Raf	Activated Raf
PTEN	Phosphatase and tensinhomolog deleted on chromosome ten
pPTEN	Phosphorylated PTEN
AKT	AKT protein
2C4	Pertuzumab
MAPK	Mitogen-activated protein kinase
MEK	MAPK/ERK kinase
pMEK	Phosphorylated MEK
ppMEK	Doubly phosphorylated MEK
ERK	extracellular signal-regulated kinase
pERK	Phosphorylated ERK
ppERK	Doubly phosphorylated ERK
MKP3	MAPK phosphatase 3
PDK1	3-phosphoinositide-dependent kinase 1
PI	Phosphatidylinositol
PIP3	phosphatidylinositol-3,4,5-trisphosphate
PI3K	phosphatidylinositol 3'-kinase
PI3K	Activated PI3K
PP2A	protein phosphatase 2A
Shc	Srchomology and collagen domain protein
pShc	Phosphorylated Shc
Sos	Son of Sevenless homolog protein
GS	Grb2–Sos complex
pE23H-Shc	Complex of pE23H with Shc
pE23H-pShc	Complex of pE23H with pShc
Shc-GS	Complex of Shc with GS
pE23H-pShc-GS	Complex of pE23H-pShc with GS
AKT-PIP3	Complex of AKT with PIP3
pAKT-PIP3	Complex of pAKT with PIP3
ppAKT-PIP3	Complex of ppAKT with PIP3

Table 1. ***Cont.***

**Abbreviations in ODEs**	**Protein names**
ppAKT-PIP3-PP2A	Complex of ppAKT-PIP3 with PP2A
AKT-PIP3-PP2A	Complex of AKT-PIP3 with PP2A
pAKT-PIP3-PP2A	Complex of pAKT-PIP3 with PP2A
E2-2C4	Complex of E2 with 2C4
E2-2C4$_c$	Complex of E2 with 2C4
PTEN-PTEN	Complex of PTEN with PTEN
pPTEN-PTEN	Complex of pPTEN withPTEN
PTEN-PIP3	Complex of PTEN with PIP3
PTEN-PI	Complex of PTEN with PI
pMEKP-PP2A	Complex of pMEKP with PP2A
MEK-PP2A	Complex of MEK with PP2A
ppMEKPP-PP2A	Complex of ppMEKPP with PP2A
pE23H-PI3K	Complex of pE23H with PI3K
PI3K -PI	Complex of PI3K with PI
pE23H-PI3K	Complex of pE23H with PI3K
E_{Raf}	Phosphatase dephosphorylating Raf
bpV	Bisperoxovanadium compound, bpV(pic), PTEN inhibitor
LY	LY294002, PI3K inhibitor
PTEN-bpV	Enzyme-inhibitor complex of PTEN and bpV(pic)
PI3K-LY	Enzyme-inhibitor complex of PI3K and LY294002
E22	HER2 homodimer
E2-Tr	HER2-trastuzumab complex
E2-Per	HER2-pertuzumab complex
E22-Per2	HER2-pertuzumab- HER2-pertuzumab complex
E2-Tr-Per	HER2-pertuzumab- HER2- trastuzumab complex

Table 2. Kinetic parameters of the model. k_i , k_{-i}– rate constants of forward and reverse reactions ($nM^{-1} min^{-1}$, min^{-1}); $K_{d,i}$, $K_{m,i}$ – dissociation and Michaelis constants (nM).

Reaction Rate	Kinetic Parameters	Values in Our Model	Remarks	Values from [1]	Values from [2]
		**HER3 and HER2 binding**			
V_1	k_1	0.005	estimation	0.001	0.003
	$K_{d,1}$	600; 0.2[1)]	estimation	0.6	20
V_{51}	k_{51}	0.01	estimation		
V_2	k_2	10	estimation	0.01	0.01
V_{52}	$K_{d,2}$	10	estimation	10	10
V_{53}	k_{53}	0.01	estimation		
V_3	k_3	1	estimation	1	1
	$K_{d,3}$	0.1	estimation	0.01	0.01
V_4	$V_{max,4}$	10	estimation	62	450
	$K_{m,4}$	50	estimation	50	50
		**Shc and GS binding**			
V_5	k_5	0.06	estimation	0.1	0.09
	$K_{d,5}$	1	estimation	1	6
V_6	k_6	12	estimation	20	6
	$K_{d,6}$	3	estimation	5	0.06
V_7	k_7	36	estimation	60	0.009
	$K_{d,7}$	9	estimation	9	4.3
V_8	k_8	12	estimation	2040	0.12
	$K_{d,8}$	0.1	estimation	7.8	0.002
V_9	k_9	35	estimation	40.8	0.1
	k_{-9}	0	estimation	0	0.2
V_{10}	$V_{max,10}$	0.0154	estimation	0.0154	1.7
	$K_{m,10}$	340	estimation	340	340
		**E23HP binding with PI3K and PI3K activation**			
V_{27}	k_{27}	3	estimation	0.1	
	$K_{d,27}$	1	estimation	20	
V_{28}	k_{28}	300	estimation	9.85	
	k_{-28}	0	estimation	0.1	
V_{29}	k_{29}	13500	estimation	45.8	
	k_{-29}	0	estimation	0.047	
V_{30}	V_{30}	900	estimation	$V_{m,26}$=2620	

Table 2. ***Cont.***

Reaction Rate	Kinetic Parameters	Values in Our Model	Remarks	Values from [1]	Values from [2]
		**Ras/Raf/MEK/ERK cascade**			
V_{11}	k_{11}	6	estimation	0.22	
	$K_{m,11}$	0.18	estimation	0.18	
V_{12}	$V_{max,12}$	3	estimation	0.3	
	$K_{m,12}$	0.1	estimation	0.06	
V_{13}	k_{13}	1	estimation	1.53	
	$K_{m,13}$	11.7	estimation	11.7	
V_{14}	k_{14}	0.6	estimation	$6.7\ 10^{-3}$	
	$K_{m,14}$	30	estimation	8	
$V_{15}\ V_{19}$	k_{15}	2.1	estimation	3.5	
	$K_{m,15}$	1	estimation	317	
V_{16}	k_{16}	0.06	estimation		
V_{17}, V_{21}	$k_{cat,16}$	0.6	estimation		
V_{18}	k_{18}	3	estimation		
V_{20}	k_{16}	0.06	estimation		
	$K_{d,16}$	1	estimation		
V_{22}	k_{22}	0.06	estimation		
$V_{23}\ V_{25}$	k_{23}	1.2	estimation	9.5	
	$K_{m,23}$	10	estimation	$1.4\ 10^{5}$	
$V_{24}\ V_{26}$	$V_{max,24}$	1.8	estimation	0.3	
	$K_{m,24}$	10	estimation	160	
		**PIP3 $\rightarrow$ PI**			
V_{31}	k_{31}	0.03	estimation		
	$K_{d,31}$	140	estimation	K_m =40	
V_{55}	k_{55}	30	estimation	k_{27} =16.9	
V_{56}	k_{56}	30	estimation		
V_{32}	k_{32}	8000	estimation		
	$K_{d,32}$	0.01	estimation	$K_{m,28}$=9	
V_{33}	k_{33}	15±5	estimation		
V_{34}	k_{34}	3.6	estimation		
		**PTEN $\rightarrow$ pPTEN**			
V_{35}	$V_{max,35}$	150	estimation		
	$K_{m,35}$	2	estimation		
V_{36}	k_{36}	1	estimation		
	$K_{d,36}$	2.2	estimation		
V_{37}	k_{37}	150	estimation		
V_{38}	k_{38}	150	estimation		

Table 2. ***Cont.***

Reaction Rate	Kinetic Parameters	Values in Our Model	Remarks	Values from [1]	Values from [2]
**AKT activation pathway**					
V_{39}	k_{39}	15000	estimation	507	
	$K_{d,39}$	20	estimation	234	
V_{40} V_{44}	$V_{max,40}$	$1.5 \cdot 10^4$	estimation	$2 \cdot 10^4$	
	$K_{m,40}$	0.1	estimation	$8 \cdot 10^4$	
V_{41}	k_{41}	3	estimation		
V_{42}, V_{46}	k_{42}	45	estimation		
V_{43}	k_{43}	30	estimation		
V_{45}	k_{41}	3	estimation		
	$K_{d,41}$	0.1	estimation		
V_{47}	k_{47}	0.3	estimation		
**Receptor internalization**					
V_{48}	k_{48}	0.001	estimation	0.001	
**2C4 binding with HER2**					
V_{49}	k_{49}	0.003	estimation		
	$K_{d,49}$	$2 \cdot 10^4$; 7[1)]	estimation, K_d=8.5 nM [3]		
V_{50}	k_{50}	0.6	estimation		
	k_{-50}	0.012	estimation		
**PTEN inhibition by bpV(pic)**					
V_{57}	k_{57}	100	estimation		
	$K_{d,57}$	10	estimation IC_{50}=31 nM [4]		
**PI3K inhibition by LY294002**					
V_{58}	k_{58}	100	estimation		
	$K_{d,58}$	80	estimation, K_i=1600 nM [5]		
**HER2 homodimerisation**					
V_{59}	k_{59}	1	estimation		
	$K_{d,59}$	100	estimation		
**Trastuzumab binding with HER2**					
V_{65}	k_{60}	0.003			
	$K_{d,60}$	$2 \cdot 10^4$; 7[1)]			

[1)] the value of the parameters after rescaling of HRG and 2C4, trastuzumab concentrations to extracellular volume [2].

Table 3. Initial concentrations of the metabolites in the model, nM.

Species	Values in the model	Remarks	Values in the model [1]	Values in the model [2]
HER3	80	estimation	HER4 = 80	EGFR = 100
HER2	50 for MCF7 cells<br>800 for SKOV3 cells	estimation		
HRG	100	estimation	100	
Shc	100	estimation	100	150
GS	100	estimation	10	
RasGDP	120	estimation	120	
Raf	100	estimation	100	
E_{Raf}	7	estimation	7	
MEK	100	estimation	120	
ERK	100	estimation	1000	
PI3K	200	estimation	10	
AKT	100	estimation	10	
PP2A	10	estimation	11.4	
PI	300	estimation	800	
PTEN	50	estimation		

References

1. Hatakeyama, M.; Kimura, S.; Naka, T.; Kawasaki, T.; Yumoto, N.; Ichikawa, M.; Kim, J.H.; Saito, K.; Saeki, M.; Shirouzu, M.; *et al.* A computational model on the modulation of mitogen-activated protein kinase (MAPK) and Akt pathways in heregulin-induced ErbB signalling. *Biochem. J.* **2003**, *373*, 451–463.
2. Kholodenko, B.N.; Demin, O.V; Moehren, G.; Hoek, J.B. Quantification of short term signaling by the epidermal growth factor receptor. *J. Biol. Chem.* **1999**, *274*, 30169–30181.
3. Adams, C.W.; Allison, D.E.; Flagella, K.; Presta, L.; Clarke, J.; Dybdal, N.; McKeever, K.; Sliwkowski, M.X. Humanization of a recombinant monoclonal antibody to produce a therapeutic HER dimerization inhibitor, pertuzumab. *Cancer Immunol. Immunother.* **2006**, *55*, 717–727.
4. Schmid, A.C.; Byrne, R.D.; Vilar, R.; Woscholski, R. Bisperoxovanadium compounds are potent PTEN inhibitors. *FEBS Lett.* **2004**, *566*, 35–38.
5. Vlahos, C.J.; Matter, W.F.; Hui, K.Y.; Brown, R.F. A specific inhibitor of phosphatidylinositol 3-kinase, 2-(4-morpholinyl)-8-phenyl-4H-1-benzopyran-4-one (LY294002). *J. Biol. Chem.* **1994**, *269*, 5241–5248.
